# WTAP-mediated m^6^A modification of lncRNA NORAD promotes intervertebral disc degeneration

**DOI:** 10.1038/s41467-022-28990-6

**Published:** 2022-03-18

**Authors:** Gaocai Li, Liang Ma, Shujie He, Rongjin Luo, Bingjin Wang, Weifeng Zhang, Yu Song, Zhiwei Liao, Wencan Ke, Qian Xiang, Xiaobo Feng, Xinghuo Wu, Yukun Zhang, Kun Wang, Cao Yang

**Affiliations:** 1 Department of Orthopaedics, Union Hospital, Tongji Medical College, Huazhong University of Science and Technology, 430022 Wuhan, China; 2 Department of Cardiology, Union Hospital, and Key Laboratory of Biological Targeted Therapy of the Ministry of Education, Tongji Medical College, Huazhong University of Science and Technology, 430022 Wuhan, China

**Keywords:** Senescence, Epigenetics, Mechanisms of disease, Long non-coding RNAs, RNA modification

## Abstract

N6-methyladenosine (m^6^A) is the most prevalent RNA modification at the posttranscriptional level and involved in various diseases and cellular processes. However, the underlying mechanism of m^6^A regulation in intervertebral disc degeneration (IVDD) remains elusive. Here, we show that methylation of the lncRNA NORAD significantly increases in senescent nucleus pulposus cells (NPCs) by m^6^A sequencing. Subsequent loss- and gain-of-function experiments reveal WTAP is increased in senescent NPCs due to an epigenetic increase in H3K4me3 of the promoter mediated by KDM5a, and significantly promotes NORAD m^6^A modification. Furthermore, YTHDF2-mediated decay of NORAD is enhanced in senescent NPCs, and then deficiency of NORAD results in less sequestraion of PUMILIO proteins, contributing to the augmented activity of PUM1/2, thus repressing the expression of target E2F3 mRNAs and promoting the cellular senescence. Here, we show interruption of NORAD m^6^A modification or the NORAD/PUMILIO/E2F3 axis could serve as a potential therapeutic target to inhibit the senescence of NPCs and development of IVDD.

## Abstract (web summary)

Intervertebral disc degeneration (IVDD) is the leading cause of low back pain and Nucleus pulposus cell senescence contributes a lot to its progression. Here, the authors reveal WTAP-mediated m6A modification of lncRNA NORAD promotes IVDD.

## Introduction

Low back pain is the leading condition contributing to the global economic burden and intervertebral disc degeneration (IVDD) is the most prevalent degenerative disease associated with low back pain^1^. During the degeneration process, the vitality of aged nucleus pulposus cells (NPCs) and the hydration and proteoglycan content of the inner nucleus pulposus (NP) decrease significantly, leading to degeneration of the NP and intervertebral disc^2–4^. Senescent NPCs exhibit decreased cellular function, resulting in the downregulation of growth factors and changes in the disc microenvironment^5,6^. However, the specific mechanism underlying the senescence of NPCs remains elusive.

Aging-associated disorders of multiple tissues and organs are accompanied by epigenetic alterations, including chromatin remodeling, histone modification, DNA methylation, and noncoding RNA regulation^7^. Histone modifications and chromatin accessibility regulation are important in the context of ageing^8,9^. DNA methylation levels are altered during aging, and these alterations can be used to predict chronological age in a variety of organs^10,11^. Furthermore, recent studies have demonstrated that microRNAs, lncRNAs, and circRNAs have critical functions in modulating aging kinetics as additional layers of epigenetic regulation^12–16^. However, as one of the most strongly aging-associated diseases, investigation into the epigenetic changes of IVDD is really limited. Thus, it is critical to explore the distinct epigenetic mechanisms during NPC senescence for a better understanding of IVDD pathogenesis, which will contribute to the development of potentially effective therapeutic strategies.

Histone modification plays a critical regulatory role in gene expression by facilitating or blocking chromatin openness and transcription^17^. Alteration of histone modification has been implicated in the initiation and development of various diseases, including sarcoma, leukemia, renal aging, and autoimmune diseases^18–21^. Furthermore, recent studies revealed crosstalk between histone modifications and other epigenetic mechanisms, such as noncoding RNAs, DNA methylation, and chromatin remodeling, as a crucial regulator of gene expression, illuminating a deeper layer of epigenetic regulation^22–24^. However, the functional role of histone modification and the interaction with epigenomics during IVDD remains poorly understood.

N6-methyladenosine (m^6^A) is the most prevalent RNA modification in eukaryotic cells at the posttranscriptional level and participates in various biological and pathological processes^25^. The m^6^A modification is catalyzed by “writers”, including METTL3, METTL14, and WTAP, which form a methyltransferase complex that adds a methyl group to the N6 site of adenine in the RRACU sequence motif (R refers to G or A), while the demethylases ALKBH5 and FTO function as “erasers” that reverse this process^26–28^. When modified with m^6^A, transcripts can be recognized by “readers”, such as YTHDF1-3, YTHDC1, and IGF2BPs, to execute a corresponding regulatory function^29,30^. Binding YTHDF2 targets m^6^A-containing RNAs and promotes their degradation, while IGF2BPs recognition increases the stability of mRNAs and promotes translation efficiency^25,30^. Moreover, the m^6^A modification of noncoding RNAs can also affect their function by regulating the fate and turnover of the transcripts^31^. However, whether the m^6^A epigenetic modification of lncRNAs participates in the aging process of NPCs remains unknown.

In this study, we investigated m^6^A modification during IVDD and NPC senescence. We found that WTAP, the core of the methyltransferase complex, was upregulated by the epigenetic alteration of H3K4me3 modification of the promoter mediated by KDM5a and lncRNA NORAD could be methylated by WTAP, resulting in the senescence of NPCs by decreasing the number of sequestered Pumilio (PUM)1/2 proteins, subsequently increasing the degradation of the transcription factor E2F3 mediated by PUM1/2. These findings suggest that the WTAP/NORAD/PUMILIO/E2F3 axis regulated by H3K4me3 could be a potential target to improve and treat IVDD.

## Results

### Nucleus pulposus aging is accompanied by NPC senescence during IVDD

**Fig. 1:**
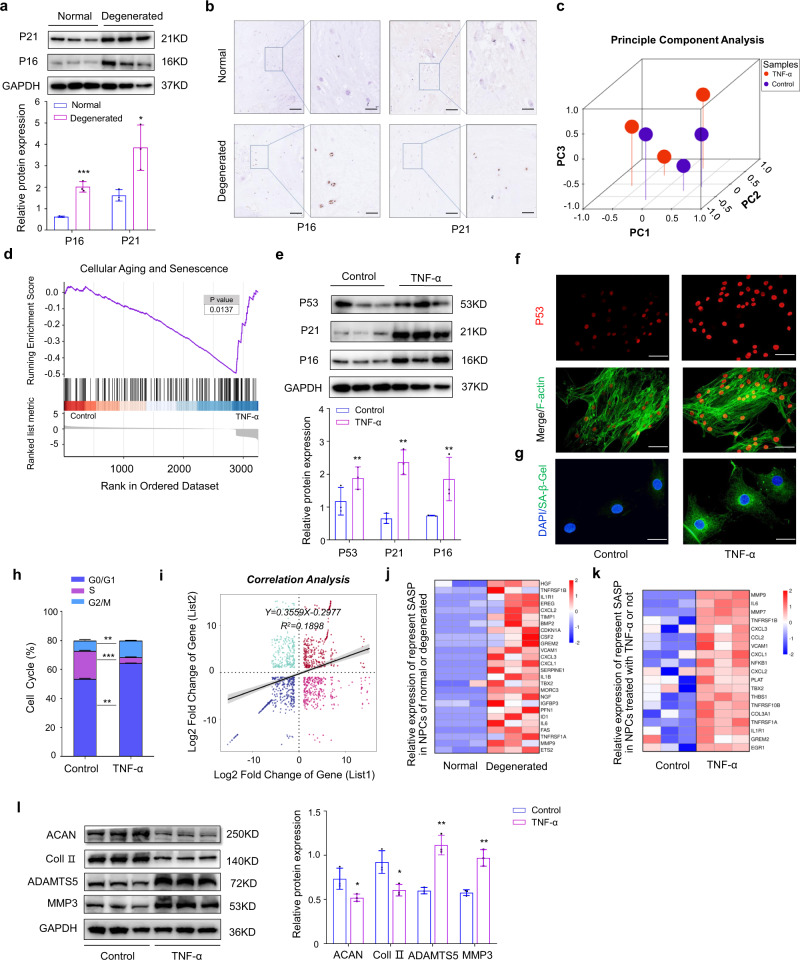
Nucleus pulposus aging is accompanied by NPC senescence during IVDD. **a** Protein level analysis of P16 (*n* = 3, *P* = 0.0006) and P21 (*n* = 3, *P* = 0.024) in normal and degenerated NP tissues by western blot, GAPDH was used as a loading control.; *P < 0.05, ***P < 0.001, two-tailed unpaired Student’s *t* test, Data are presented as mean ± SD. **b** IHC analysis of P16 and P21 expression in normal and degenerated NP tissues (scale bar: 100 μm, 20 μm), three independent experiments were repeated. **c** Principal component analysis (PCA) of samples using for sequencing. **d** GSEA analysis of sequencing data of NPCs treated with TNF-α or not. The two-sided Fisher’s exact test with adjustments was made for multiple testing. **e** Protein level analysis of P53 (*P* = 0.0016), P16 (*P* = 0.0019) and P21 (*P* = 0.0020) in normal and senescent NPCs (TNF-α) by western blot, GAPDH was used as the loading control. Data are shown as the mean ± SD, *n* = 3. **P < 0.01, two-tailed unpaired Student’s *t* test. **f**, **g** IF analysis of P53 expression (red: P53; green: F-actin; scale bar: 50 μm) and SA-β-gal activity (green: SA-β-gal; blue: DAPI; Scale bar: 20 μm) in normal and senescent NPCs, three independent experiments were repeated. **h** Cell cycle analysis of NPCs treated with or without TNF-α. **P < 0.01, ***P < 0.001, two-tailed unpaired Student’s *t* test, *n* = 3, Data are presented as mean ± SD. **i** Correlation analysis of sequencing data of transcripts in cultured NPCs (list2) with transcripts in the NPCs from human discs (list1). Data are presented as mean ± SD. **j**, **k** Separate heatmap of represent different SASP expression in sequencing data of normal and senescent NPCs or NPCs from normal or degenerated NP tissues. **l** Protein level analysis of ACAN (*P* = 0.0425), Col II (*P* = 0.0191), ADAMTS5 (*P* = 0.0016), and MMP3 (*P* = 0.0024) in normal and senescent NPCs by western blot, GAPDH was used as the loading control. Data are shown as the mean ± SD from three independent experiments. *P < 0.05, **P < 0.01, two-tailed unpaired Student’s *t* test.

Previous studies reported that IVDD is accompanied by NP degeneration and NPC senescence^32,33^. To further explore the mechanism of NP aging in the degeneration process, we analyzed the expression of aging-associated markers in NP tissues and verified that expression of these markers in degenerated NP tissues was higher than in normal tissues (Fig. 1a, b). Subsequently, TNF-α was administered to establish an in vitro model of NPC degeneration and RNA sequencing was performed to further explore the underlying pathological mechanism of NP degeneration^16^. Principle component analysis showed that NPCs treated with TNF-α clustered apart from the corresponding normal NPCs (Fig. 1c). Next gene set enrichment analysis (GSEA) showed that treatment of the NPCs with TNF-α was accompanied with cellular aging and senescence (Fig. 1d), which was fruther evidenced by increased expression of senescence-associated markers, indicated by western blot, immunofluorescence, and SA-β-gal activity analysis (Fig. 1e–g). Meanwhile, cell cycle analysis showing a decreased percentage of cells in the synthesis (S) phase after TNF-α treatment further verified these results (Fig. 1h). NPCs from human degenerated and normal discs were then isolated and transcripts were used to perform next-generation sequencing and data analysis showed that the change in the expression pattern of transcripts from degenerated human discs was consistent to some extent with that of NPCs cultured in vitro with TNF-α (Fig. 1i). We mapped the differentially expressed represent senescence-associated genes (SASP), and we observed a clear similarity between the differentially expressed genes (DEGs) in NPCs from degenerated NP tissues versus those NPCs from normal discs, and between TNF-α-treated NPCs versus control cells (Fig. 1j, k). Moreover, as shown by western blot (Fig. 1l), the expression of collagen II and aggrecan in NPCs after TNF-α treatment declined, while both MMP3 and ADAMTS5 increased significantly. The above data suggest that the senescent status of NPCs during IVDD correlated with that of NPCs cultured with TNF-α.

### m^6^A modification of the lncRNA NORAD contributes to NPC senescence

**Fig. 2:**
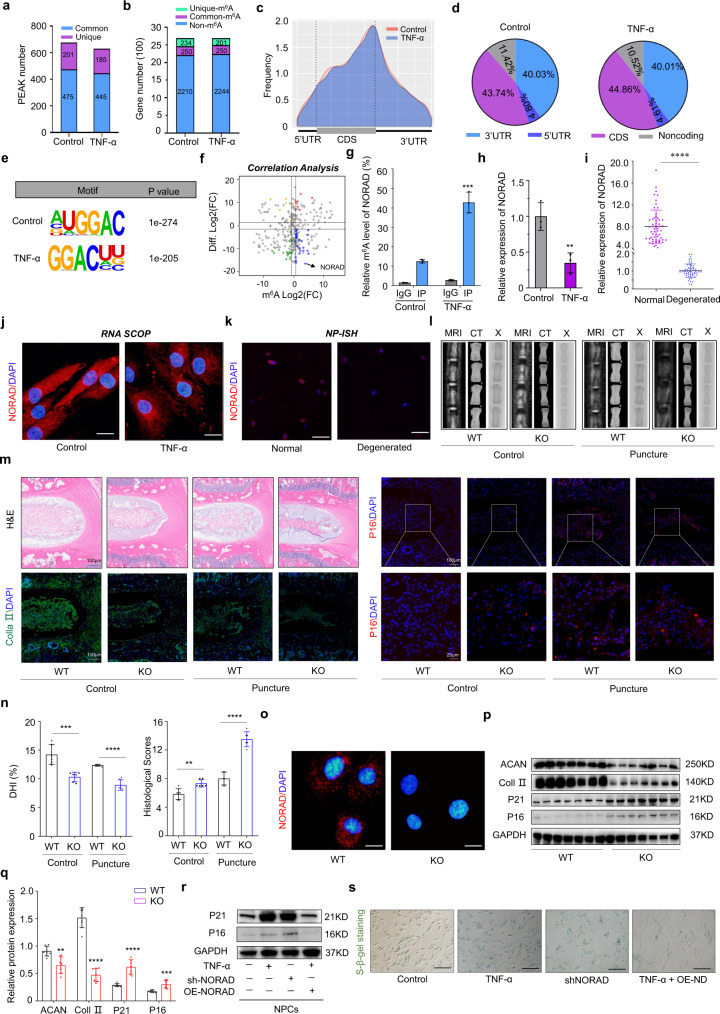
m^6^A modification of the lncRNA NORAD contributes to NPC senescence. **a**, **b** PEAK and gene number analysis of Me-RIP-Seq results of NPCs. **c**, **d** PEAK distribution of gene regions of m^6^A-modified transcripts. **e** Representative motif of m^6^A modification of transcripts in normal and senescent NPCs, two-sided Fisher’s exact test with adjustments for multiple testing. **f** Correlation analysis of m^6^A and transcript-differ genes. **g** m^6^A level of NORAD transcripts in normal and senescent NPCs by Me-RIP-qPCR. Data are shown as the mean ± SD, *n* = 3, *P* = 0.0007, *P < 0.05, two-tailed unpaired Student’s *t* test. **h**, **i** Expression of NORAD in NPCs (*n* = 3, *P* = 0.0094) and NP tissues (*n* = 58, *P* < 0.0001) by RT-qPCR. Data are shown as the mean ± SD, **P < 0.01, ****P < 0.0001, two-tailed unpaired Student’s *t* test. **j**, **k** NORAD expression in NPCs and NP tissues by FISH (red: NORAD; blue: DAPI; scale bar: 20 μm; 50 μm). **l** Radiographic presentation of control and IVDD model by MRI, μCT and X-Ray. **m** Histological of IVDD model analyzed by HE staining and IF analysis (green: Collagen II; red: P16; blue: DAPI; scale bar: 100 μm; 25 μm). **n** Radiographic analysis of IVDD model by DHI% in control (*P* = 0.0005) and puncture group (*P* < 0.0001); *n* = 6, ***P < 0.001, ****P < 0.0001. Histological score of IVDD model in control (*P* = 0.0024) and puncture group (*P* < 0.0001); two-tailed unpaired Student’s *t* test. *n* = 6, **P < 0.01, ****P < 0.0001, two-tailed unpaired Student’s *t* test. **o** NORAD expression in NPCs from WT and KO mice by FISH (red: NORAD; blue: DAPI; scale bar: 20 μm). **p**, **q** Protein level of ACAN (*P* = 0.0022), Collagen II (*P* < 0.0001), P16 (*P* < 0.0001), P21 (*P* = 0.0005) in NPCs from WT and KO mice by western blot, GAPDH was used as a loading control. Data are shown as the mean ± SD, *n* = 7. **P < 0.01, ***P < 0.001, ****P < 0.0001, two-tailed unpaired Student’s *t* test. **r** Protein level of P16 and P21 in NPCs with knockdown or overexpression of NORAD (sh-ND: shRNA of NORAD; OE-ND: overexpression of NORAD); GAPDH was used as a loading control. **s** SA-β-gal staining of NPCs with knockdown or overexpression of NORAD (Scale bar: 200 μm).

To further explore whether m^6^A modification of lncRNAs influences the senescence of NPCs, methylated RNA immunoprecipitation sequencing (Me-RIP-Seq) was performed to analyze lncRNAs with different m^6^A peaks accompanied by different levels of the transcripts. In total, m^6^A-seq identified 676 and 630 m^6^A peaks from 484 to 451 m^6^A-modified transcripts in control and senescent NPCs respectively (Fig. 2a, b). Peak distribution analysis showed that m^6^A sites were enriched in both exons and 3’UTRs, with the highest enrichment of m^6^A residues located near the stop codon, which was in line with previous analyses (Fig. 2c, d). The consensus motif of RRACU (R = A, G) was enriched in m^6^A-purified peaks (Fig. 2e). Correlation analysis showed that m^6^A modification of the lncRNA NORAD was significantly increased in senescent NPCs, while transcript expression was decreased, as verified by the results of Me-RIP-qPCR and RT-qPCR (Fig. 2f–h), in accordance with the results of fluorescence in situ hybridization (FISH) analysis of NORAD transcripts (Fig. 2j). Additionally, downregulated NORAD expression was further confirmed by RT-qPCR and RNA scope ISH in normal and degenerated NP tissues (Fig. 2i, k). To explore the critical role of NORAD in IVDD, we constructed *NORAD* knockout mice using CRISPR/Cas9-mediated genome editing technology, which showed a degenerative phenotype resembling premature aging and displayed pronounced kyphosis, resembling aged humans with degenerated discs and bones, consistent with previous report^34^ (Supplementary Fig. 1a–d). Histological analysis of IVD showed degeneration occurred in KO mice at age of 2 months, manifested by collapsed disc space, narrowed endplates height and a moderate decrease in the cellularity of the nucleus pulposus (Fig. 2l). To further elucidate the role of NORAD in the development of IVDD, a surgically induced IVDD model was established in WT and KO mice for two months. Degenerative change of NPs was more significant in IVD punctured groups, where more severe degeneration of NPs and IVD was observed in KO mice (Fig. 2l–n). These results were further confirmed by radiographic and histological assessments, showing progressive disc space narrowing, worse MRI manifestation, and markedly decreased histologic scores in KO punctured groups. The above results demonstrate that NORAD deficiency not only contributes to spontaneous IVDD initiation and progression, but accelerates surgically induced IVDD in mice (Fig. 2l–n). Further cellular analysis of NPCs isolated from WT and KO mice revealed deletion of NORAD promotes the senescence of NPCs using FISH and western blot (Fig. 2o–q). Moreover, gain- and loss-of-function experiments were performed in human NPCs, and results demonstrated that NORAD deficiency with aging contributed to cellular senescence, while the restoration of NORAD ameliorated the senescent phenotype induced by TNF-α to some extent (Fig. 2r, s).

**Fig. 3:**
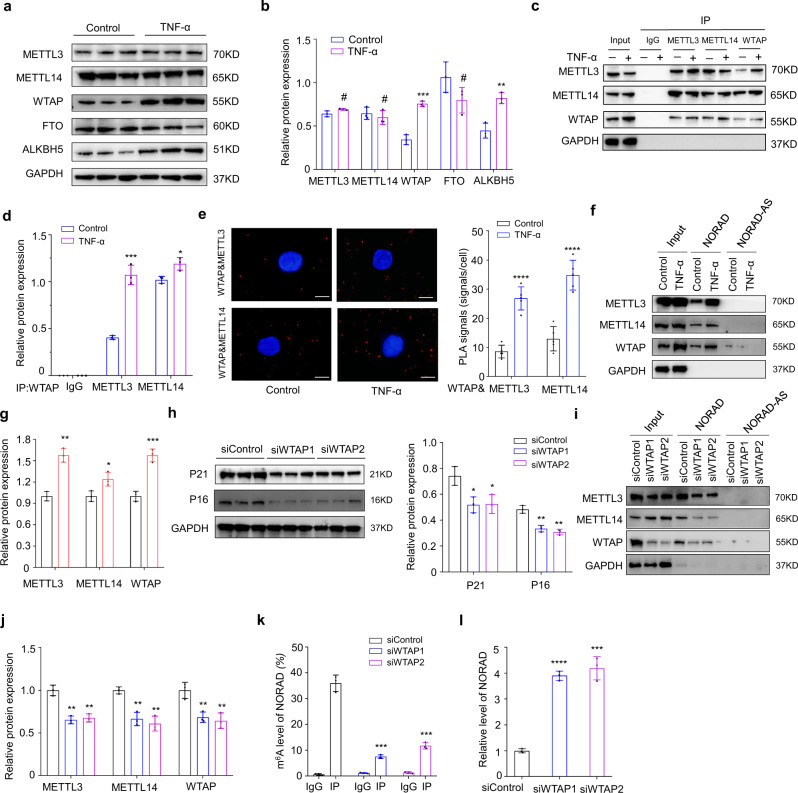
m^6^A modification of the lncRNA NORAD contributes to NPC senescence. **a**, **b** Protein level of m^6^A METTL3 (*P* = 0.0673), METTL14 (*P* = 0.4789), WTAP (*P* = 0.0004), FTO (*P* = 0.1135), and ALKBH5 (*P* = 0.0036) in normal and senescent NPCs by western blot, GAPDH was used as the loading control. Data are shown as the mean ± SD, *n* = 3. ^#^NS, *P < 0.05, ***P < 0.001, two-tailed unpaired Student’s *t* test. **c**, **d** Co-IP analysis of the interaction between WTAP and METTL3 (*P* = 0.0004), METTL14 (*P* = 0.0180). Data are presented as mean ± SD, *n* = 3. *P < 0.05, ***P < 0.001, two-tailed unpaired Student’s *t* test. **e** PLAs were used to demonstrate the physical interaction between WTAP and METTL3 (*P* < 0.0001) or METTL14 (*P* < 0.0001) (red: signal; blue: DAPI; scale bar: 20 μm). Data are presented as mean ± SD, *n* = 5, *P < 0.05, ****P < 0.0001, two-tailed unpaired Student’s *t* test. **f**, **g** RNA pulldown of NORAD with METTL3 (*P* = 0.001), METTL14 (*P* = 0.0277), WTAP (*P* = 0.0007) in NPCs treated with TNF-α or not. Data are shown as the mean ± SD, *n* = 3. *P < 0.05, **P < 0.01,***P < 0.001, two-tailed unpaired Student’s *t* test. **h** Protein level of P21 (*P* = 0.0252; 0.0219) and P16 (*P* = 0.0027; 0.0012) in TNF-α-treated NPCs by western blot with WTAP silencing or not, GAPDH was used as the loading control. Data are shown as the mean ± SD from three independent experiments. *P < 0.05, **P < 0.01, two-tailed unpaired Student’s *t* test. **i**, **j** RNA-pulldown of NORAD with METTL3 (*P* = 0.0027; 0.0022), METTL14 (*P* = 0.0084; 0.0094), WTAP (*P* = 0.0014; 0.0020) in NPCs with WTAP silencing or not, followed by western blot. Data are shown as the mean ± SD, *n* = 3. **P < 0.01, two-tailed unpaired Student’s *t* test. **k** Me-RIP-qPCR analysis of the m^6^A level of NORAD transcripts (*P* = 0.0001; 0.0002) in NPCs with or without WTAP silencing. Data are shown as the mean ± SD from three independent experiments. ***P < 0.001, two-tailed unpaired Student’s *t* test. **l** RT-qPCR analysis of the expression of NORAD in NPCs (*P* < 0.0001; *P* = 0.0003) with or without WTAP silencing. Data are shown as the mean ± SD, *n* = 3, ***P < 0.001, ****P < 0.0001, two-tailed unpaired Student’s *t* test.

To explicate the regulatory mechanism of the m^6^A modification, the expression of methylases and demethylases in NPCs were analyzed by western blot analysis, the results of which showed that expression of the methylase WTAP was significantly increased, in accordance with the sequencing results (Fig. 3a, b and Supplementary Fig. 2a). In addition, the overall m^6^A modification on total mRNA was significantly upreguated in senescent NPCs (Supplementary Fig. 2b, c). The significant increase of WTAP expression may account for the hypermethylation of NORAD, while upregulation of ALKBH5 may contribute to the opposite effect. Co-immunoprecipitation analysis showed that more METTL3 and METTL14 interacted with WTAP to form methyltransferase complexes in senescent NPCs, which might induce the hypermethylation of NORAD (Fig. 3c, d). To elucidate the status of the methyltransferase complex more clearly, we analyzed the binding status of WTAP to METTL3 and METTL14. Proximity ligation assays (PLAs) were employed and results showed that interactions between WTAP and METTL3/METTL14 were more prominent in senescent NPCs (Fig. 3e). Furthermore, RNA-pulldown experiments followed by western blot were performed to indicate increased WTAP in senescent NPCs promoted the interaction between NORAD and methyltransferase complex, including METTL3 and METTL14 (Fig. 3f, g). Additionally, WTAP was silenced in human NPCs by two separate siRNAs (Supplementary Fig. 2d), which could alleviate the senescence status of NPCs treated with TNF-α (Fig. 3h). Moreover, knockdown of WTAP abolished the interaction of NORAD with METTL3 and METTL14 (Fig. 3i, j) and abrogated the hypermethylation of NORAD while partially rescued its expression in NPCs treated with TNF-α (Fig. 3k, l). These results demonstrated that WTAP-mediated m^6^A modification of NORAD contributed to NPC senescence.

### WTAP upregulation is induced by the epigenetic alteration of H3K4me3

**Fig. 4:**
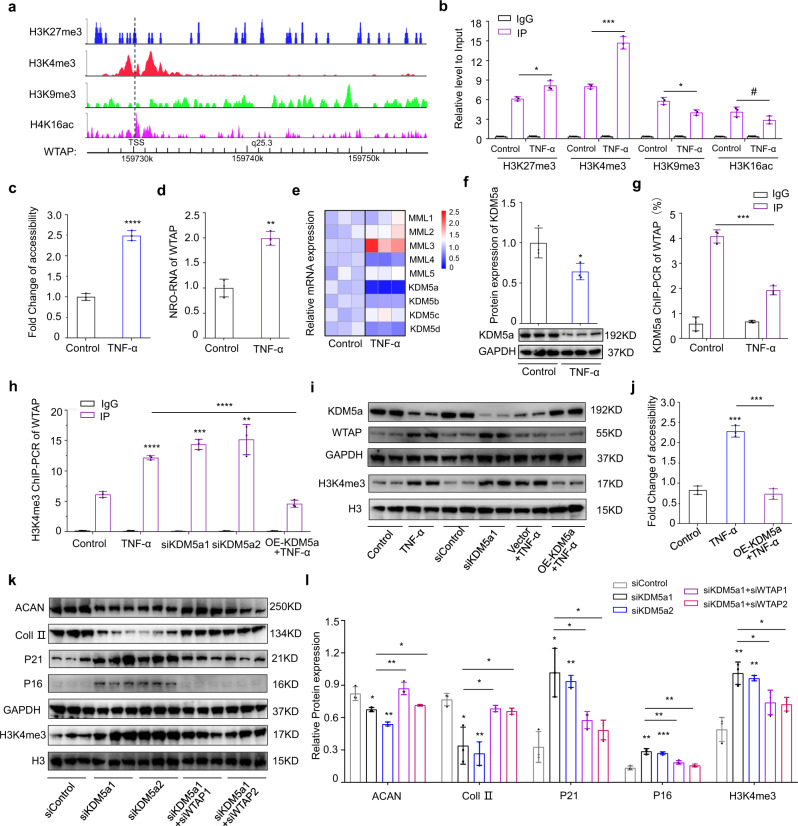
WTAP upregulation is regulated by the epigenetic alteration of H3K4me3. **a** Prediction of Histone modification of the WTAP promoter using the WashU Epigenome Browser. **b** ChIP-PCR analysis of H3K27me3 (*P* = 0.0106), H3K4me3 (*P* = 0.0003), H3K9me3 (*P* = 0.0102) and H4K16ac (*P* = 0.0888) modification in the promoter of WTAP. Data are shown as the mean ± SD, *n* = 3, ^#^NS, *P < 0.05, ***P < 0.001, two-tailed unpaired Student’s *t* test. **c** DNase I sensitivity assay to analyze the chromatin accessibility of WTAP (*P* < 0.0001). Data are shown as the mean ± SD, n = 3, ****P < 0.0001, two-tailed unpaired Student’s *t* test. **d** Nuclear run-on (NRO) assays of WTAP (*P* = 0.0016) in NPCs. Data are shown as the mean ± SD, n = 3, **P < 0.01, two-tailed unpaired Student’s *t* test. **e** Heatmap of lysine methylases and demethylases of H3K4me3 by RT-qPCR. **f** Expression of KDM5a by western blot, GAPDH was used as the loading control. Data are shown as the mean ± SD, *n* = 3, *P* = 0.0435. *P < 0.05, two-tailed unpaired Student’s *t* test. **g** ChIP-PCR analysis of KDM5a interaction with WTAP promoter. Data are shown as the mean ± SD, *n* = 3, *P* = 0.0003. ***P < 0.001, two-tailed unpaired Student’s *t* test. **h** ChIP-PCR analysis of H3K4me3 modification of the WTAP promoter in TNF-α (*P* < 0.0001), siKDM5a1 (*P* = 0.0001), siKDM5a2 (*P* = 0.0035) and OE-KDM5a + TNF-α (*P* < 0.0001) groups. Data are shown as the mean ± SD, *n* = 3. **P < 0.01, ***P < 0.001, ****P < 0.0001, two-tailed unpaired Student’s *t* test. **i** Protein expression in NPCs with silencing or overexpression of KDM5a by western blot, GAPDH and H3 were used as the loading control. **j** DNase I sensitivity assay to analyze the chromatin accessibility of WTAP in senescent NPCs (*P* = 0.0001) with KDM5a overexpression (*P* = 0.0002). Data are shown as the mean ± SD, *n* = 3, ***P < 0.001, two-tailed unpaired Student’s *t* test. **k**, **l** Protein expression of ACAN (*P* = 0.0184; 0.0018; 0.0049; 0.0331), Coll II (*P* = 0.0149; 0.0021; 0.0266; 0.0335), P21 (*P* = 0.0109; 0.0024; 0.0329; 0.0191), P16 (*P* = 0.0011; 0.0005; 0.0044; 0.0015), and H3K4me3 (*P* = 0.0041; 0.0020; 0.0384; 0.0150) in NPCs with silencing KDM5a or not while WTAP was silencing or not meanwhile by western blot, GAPDH and H3 were used as the loading control. Data are shown as the mean ± SD, *n* = 3, *P < 0.05, **P < 0.01, ***P < 0.0001, two-tailed unpaired Student’s *t* test.

Histone modification, a key cellular epigenetic modification, influences gene expression by modulating chromatin accessibility and has been confirmed to be a major contributor to cellular ageing^35^. Increases in the histone modifications H4K16ac and H3K4me3, as well as decreases in H3K9me3 and H3K27me3, have been described as age-associated epigenetic marks^36^. To determine whether WTAP expression can be regulated through these epigenetic marks, we first analyzed modification of the WTAP promoter using the WashU Epigenome Browser (https://epigenomegateway.wustl.edu/)^37^. As shown in Fig. 4a, abundant signals were found in the promoter region of WTAP, suggesting that WTAP is regulated by histone modifications. Moreover, chromatin immunoprecipitation (ChIP) assays were used to measure the levels of these epigenetic modifications. The ChIP-PCR results showed that histone H3 lysine 4 (H3K4) trimethylation (H3K4me3) was most significantly increased in senescent NPCs (Fig. 4b and Supplementary Fig. 3a). Enrichment of H3K4me3 is an indicator of an open and accessible chromatin structure^38^. We further investigated the chromatin accessibility of WTAP by DNase I sensitivity assay, and observed higher chromatin accessibility accompanied with less nucleosome occupancy (Fig. 4c). Furthermore, nuclear run-on assays were employed to investigate the transcription status of WTAP and showed that senescent NPCs treated with TNF-α exhibited a significant increase in transcription (Fig. 4d). To clearly elucidate the specific mechanism, we analyzed the expression of lysine methylases and demethylases of H3K4me3, including MML1-5 and KDM5a-d, by RT-qPCR and found that expression of the demethylase KDM5a was most significantly decreased in senescent NPCs, a result further confirmed by western blot (Fig. 4e, f). ChIP-PCR assay using KDM5a antibody confirmed that less KDM5a bound the WTAP promoter during senescence (Fig. 4g). Knockdown of KDM5a using two separate siRNAs promoted H3K4me3 modification and increased the expression of WTAP in NPCs, while overexpression of KDM5a in NPCs treated with TNF-α had the opposite effects (Fig. 4h, i and Supplementary Fig. 3b). In addition, downregulation of WTAP induced by the overexpression of KDM5a in NPCs was accompanied by decreased chromatin accessibility (Fig. 4j). Moreover, deficient KDM5a-induced higher H3K4me3 modification in human NPCs contributed to NPC senescence and degeneration, which could get abolished by WTAP silencing-mediated decreased m^6^A methylation (Fig. 4k, l). Taken together, the above data further confirmed that the upregulation of WTAP was due to increased H3K4me3 alteration mediated by decreased KDM5a.

### NORAD stability is regulated by m^6^A modification

**Fig. 5:**
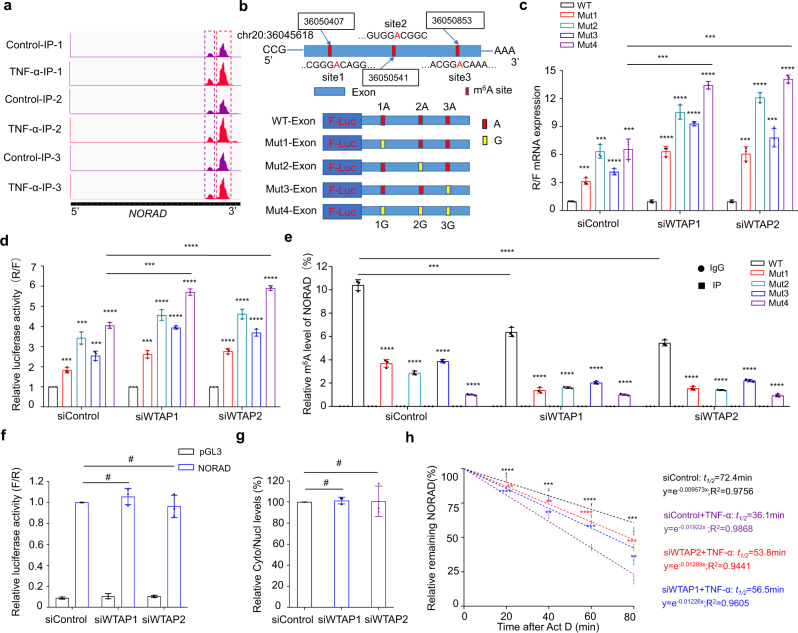
NORAD stability is regulated by m^6^A modification. **a**, **b** PEAK of NORAD transcript by Me-RIP-Seq; m^6^A sites in NORAD and the corresponding mutant sites. **c** mRNA expression of wild-type or site-mutant NPCs with or without WTAP silencing (*P* = 0.0005; 0.0004). Data are shown as the mean ± SD, *n* = 3. ***P < 0.001, ****P < 0.0001, two-tailed unpaired Student’s *t* test. **d** Dual-luciferase reporter assay of wild-type or site-mutant NPCs with or without WTAP silencing (*P* = 0.0002; *P* < 0.0001). Data are shown as the mean ± SD from three independent experiments. ***P < 0.001, ****P < 0.0001, two-tailed unpaired Student’s *t* test. **e** Me-RIP-PCR analysis of the m^6^A level of NORAD wild-type or site-mutant NPCs with or without WTAP silencing (*P* = 0.0003; *P* < 0.0001). Data are shown as the mean ± SD from three independent experiments. ***P < 0.001,****P < 0.0001, two-tailed unpaired Student’s *t* test. **f** Dual-luciferase reporter assay of the activity of the NORAD promoter in NPCs with or without WTAP silencing. Data are shown as the mean ± SD from three independent experiments. ^#^NS, two-tailed unpaired Student’s *t* test, compared between siWTAP and siControl groups. **g** Analysis of NORAD expression in nuclear and cytoplasmic fractions by RT-qPCR. Data are shown as the mean ± SD from three independent experiments. ^#^NS, two-tailed unpaired Student’s *t* test. **h** Transcript stability assay of NORAD in senescent NPCs with or without WTAP knockdown at time of 20 (*P* < 0.0001; *P* < 0.0001; *P* = 0.0018), 40 (*P* = 0.0003; *P* = 0.0041; *P* = 0.0046), 60 (*P* < 0.0001; *P* = 0.0001; *P* < 0.0001), 80 min (*P* = 0.0001; *P* = 0.0054; *P* = 0.0003). Data are shown as the mean ± SD, *n* = 3. **P < 0.01, ***P < 0.001, ****P < 0.001, two-tailed unpaired Student’s *t* test.

To understand the role of m^6^A modification in regulating NORAD expression, we analyzed transcriptome-wide m^6^A-Seq data. We found that the m^6^A peak for NORAD was enriched in exon regions ranging from sites 36,049,759 to 36,050,960, and bioinformatic analysis showed the presence of three GGACU motifs in the exon region of the NORAD transcript (Fig. 5a, b). In addition, the three adenosine sites showed significant differences in their methylation levels. Next, we generated dual-luciferase reporter constructs containing firefly luciferase before the wild-type or mutant NORAD sequence with A at the m^6^A sites substituted with G (Fig. 5b and Supplementary Fig. 4a), after which Me-RIP-qPCR was performed and showed that the site mutation decreased the m^6^A modification of NORAD while the expression level was greatly elevated (Fig. 5c–e). Meanwhile, compared with the control group, corresponding expression of NORAD in mutant NPCs with WTAP silencing were upregulated, with the m^6^A level downregulated, manifesting most significantly in all-mutant NPCs, which further confirmed the specific methylation site of NORAD (Fig. 5c–e). Next, to further determine the effect of m^6^A on NORAD, we tested the promoter activity of NORAD in control and WTAP-knockdown NPCs using luciferase reporter assays in which the promoter region of NORAD was cloned into the pGL3 luciferase reporter (Supplementary Fig. 4b). The results showed no difference in the activity of the NORAD promoter, suggesting that the NORAD transcription process was not influenced by WTAP-mediated methylation differences (Fig. 5f). Then, we separated RNAs in nuclear and cytoplasmic fractions and observed no difference in the subcellular localization of NORAD, which explains why m^6^A did not affect the export of NORAD transcripts (Fig. 5g). NPCs stimulated with TNF-α with or without WTAP silencing were treated with the transcription inhibitor actinomycin D. The level of NORAD expression was detected at the indicated time, and a half-life assay was performed. The NORAD half-life in TNF-α-stimulated cells was significantly shorter than that in control cells, while WTAP silencing reduced the effect of TNF-α treatment to some extent (Fig. 5h). These results suggest that increased m^6^A modification downregulates the stability of NORAD transcripts.

### Reduced stability of NORAD is mediated by YTHDF2

**Fig. 6:**
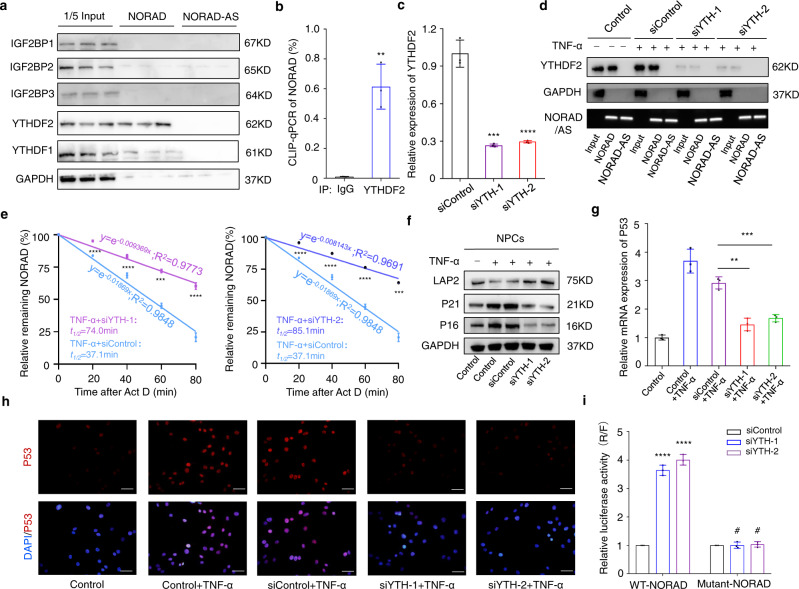
Reduced stability of NORAD is mediated by YTHDF2. **a** RNA-pulldown assay showed that YTHDF2 interacted with NORAD, three independent experiments were repeated. **b** CLIP-qPCR of YTHDF2 showed that NORAD interacted with YTHDF2. Data are shown as the mean ± SD, *n* = 3, *P* = 0.0023, two-tailed unpaired Student’s *t* test. **c** mRNA expression of NORAD in NPCs with or without YTHDF2 silencing (siYTH-1: siRNA against YTHDF2#1, *P* = 0.0003; siYTH-2: siRNA against YTHDF2#2, *P* = 0.0004). Data are shown as the mean ± SD, *n* = 3, ***P < 0.001, two-tailed unpaired Student’s *t* test. **d** RNA Pulldown assay showed the interaction of YTHDF2 with NORAD in normal or senescent NPCs with or without YTHDF2 silencing using probes of NORAD or NORAD-AS probes. **e** Stability assay of NORAD in senescent NPCs with or without YTHDF2 silencing at 20 (*P* < 0.0001; *P* < 0.0001), 40 (*P* = 0.0004; *P* < 0.0001), 60 (*P* < 0.0001; *P* < 0.0001), 80 min (*P* < 0.0001; *P* = 0.0001). Data are shown as the mean ± SD from three independent experiments. ***P < 0.001, ****P < 0.0001, two-tailed unpaired Student’s *t* test. **f** Protein level analysis of LAP2, P21 and P16 in normal and senescent NPCs with or without YTHDF2 silencing by western blot, GAPDH was used as the loading control. **g** Relative mRNA expression of P53 in normal and senescent NPCs with or without YTHDF2 silencing by RT-qPCR. Data are shown as the mean ± SD, *n* = 3, **P < 0.01, ***P < 0.001, two-tailed unpaired Student’s *t* test, comparisons between siYTH-1+TNF-α (*P* = 0.0012) or siYTH-2+TNF-α (*P* = 0.0010) and TNF-α groups. **h** IF analysis of P53 in normal and senescent NPCs with or without YTHDF2 silencing (red: P53; blue: DAPI; scale bar: 50 μm). **i** Dual-luciferase reporter assay of wild-type (*P* < 0.0001; *P* < 0.0001) or site-mutant (*P* = 0.9212; 0.5489) NPCs with or without YTHDF2 silencing. Data are shown as the mean ± SD, *n* = 3. ^#^NS, ****P < 0.0001, two-tailed unpaired Student’s *t* test.

A previous study showed that “readers”, including YTHDF2/3 and IGF2BP1/2/3, can influence the stability of m^6^A-modified RNA^39^. To identify the reader that recognizes NORAD, an RNA pulldown assay was performed, followed by western blot analysis of the isolated proteins. The results revealed that YTHDF2 interacted with NORAD, and additional CLIP-qPCR analysis confirmed that methylated NORAD strongly interacted with YTHDF2 (Fig. 6a, b), consistent with the results predicted from the RNA interaction database *RNA Intern* (Supplementary Fig. 5a). Knockdown of YTHDF2 in NPCs using two specific siRNAs decreased its recognition of methylated NORAD and rescued the expression of NORAD by reducing its function in promoting decay (Fig. 6c–e). Furthermore, YTHDF2 silencing blocked the pro-senescence effect induced by TNF-α treatment to some extent (Fig. 6f–h). Dual-luciferase reporters containing Renilla luciferase before the sequence for wild-type or mutant NORAD modified at multiple sites in control and YTHDF2-silenced NPCs showed that the expression change in YTHDF2 had no effect on mutant NORAD (Fig. 6i and Supplementary Fig. 5b). The above data implied that the decreased stability of NORAD induced by m^6^A modification was mediated by YTHDF2.

### The deficiency of NORAD contributes to NPC senescence by maintaining the PUMILIO protein

**Fig. 7:**
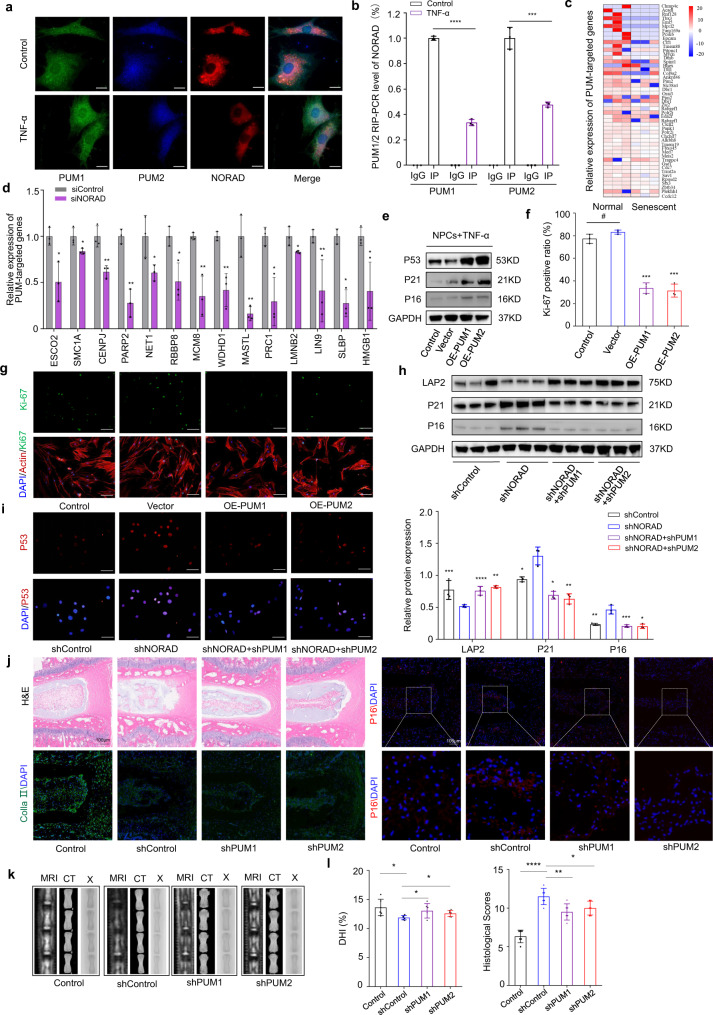
Deficiency of NORAD contributes to NPC senescence by maintaining the PUMILIO protein. **a** FISH analysis of NORAD transcripts and fluorescence immunostaining of PUM1 and PUM2 in normal or senescent NPCs (green: PUM1; blue: PUM2; red: NORAD; scale bar: 20 μm). **b** RIP-qPCR analysis of PUM1 (*P* < 0.0001) and PUM2 (*P* = 0.0005) interaction with NORAD in normal or senescent NPCs. Data are shown as the mean ± SD, *n* = 3. ***P < 0.001, ****P < 0.0001, two-tailed unpaired Student’s *t* test, compared between TNF-α and control groups. **c** Expression of PUM-targeted genes in normal and senescent NPCs by transcript sequencing shown as a heatmap. **d** Relative expression of representative PUM-targeted genes in normal and senescent NPCs by RT-qPCR. Data are shown as the mean ± SD, *n* = 3, *P* = 0.0221; 0.0463; 0.0074; 0.0019; 0.0479; 0.0204; 0.0072; 0.0056; 0.0038; 0.012; 0.0473; 0.0412; 0.0017; 0.0356. *P < 0.05, **P < 0.01, two-tailed unpaired Student’s *t* test. **e** Protein level analysis of P53, P21 and P16 in control and PUM1/2-overexpressed (OE-PUM1/2) NPCs, GAPDH was used as the loading control. **f**, **g** IF analysis of Ki67 in control (*P* = 0.0849) and PUM1/2 overexpressing (*P* < 0.0001; *P* = 0.0001) NPCs (green: Ki67; red: Actin; blue: DAPI; scale bar: 100 μm). Data are shown as the mean ± SD, *n* = 3, ^#^NS, ***P < 0.001, ****P < 0.0001, two-tailed unpaired Student’s *t* test. **h** Protein level analysis of LAP2 (*P* = 0.0009; *P* < 0.0001; *P* = 0.0029), P21 (*P* = 0.0280; *P* = 0.0446; *P* = 0.0031) and P16 (*P* = 0.0090; *P* = 0.0003; *P* = 0.0170) in control and NORAD-silenced NPCs with or without PUM1/2 knockdown, GAPDH was used as the loading control. Data are shown as the mean ± SD from three independent experiments. *P < 0.05, **P < 0.01, ***P < 0.001, two-tailed unpaired Student’s *t* test. **i** IF analysis of P53 in control and NORAD-silenced NPCs with or without PUM1/2 knockdown (red: P53; blue: DAPI; scale bar: 50 μm). **j** Histological analysis of surgically induced IVDD using AAV containing shRNA targeting PUM1/2, analyzed by HE and IF staining. **k** Radiographic presentation of IVDD model by MRI, μCT and X-Ray. **l** DHI% analysis (*P* = 0.0139; *P* = 0.0499; *P* = 0.0199) and histological score (*P* < 0.0001; *P* = 0.0080; *P* = 0.0237) of IVDD model with PUM1/2 silencing or not; Data are presented as mean ± SD, *n* = 6, **P < 0.01, ***P < 0.001; *n* = 6, *P < 0.05, **P < 0.01, two-tailed unpaired Student’s *t* test.

NORAD, which is highly conserved and more abundantly expressed than other lncRNAs, is upregulated upon DNA damage and can induce chromosomal instability when deleted^40^. Cytoplasmic lncRNAs are known to modulate the activity or abundance of interacting proteins, and a recent study reported that PUMILIO, a family of highly abundant cytoplasmic RNA-binding proteins including PUM1 and PUM2, interact with NORAD and influence the aging process^34^. Although some studies and our data suggest that the level of NORAD in the body decreases with aging, the precise regulatory mechanism involving NORAD in NPC aging remains unclear. To reveal the precise role of NORAD in the aging process, we performed FISH analysis of NORAD transcripts and fluorescence immunostaining of PUM1 and PUM2, which indicated the co-localization and interaction of NORAD and PUM1/2 in NPCs, an effect which could be attenuated by TNF-α treatment (Fig. 7a). Further RIP-qPCR assays also revealed less binding of PUM1 and PUM2 to NORAD transcripts in senescent NPCs (Fig. 7b). To validate the hypothesis that NORAD can sequester the PUMILIO protein, thus negatively regulating the ability of PUMILIO to repress target mRNAs in NPCs, we determined the expression level of representative PUMILIO target genes in transcript sequencing results^40^. The expression of these genes in senescent NPCs is shown in the heatmap (Fig. 7c). Furthermore, decreased expression of target genes were observed in siRNA-induced NORAD-deficient NPCs, manifesting hyperactivity of PUMILIO (Fig. 7d). Moreover, overexpression of PUM1 and PUM2 in wild-type NPCs promoted the senescence of NPCs, mimicking the NORAD loss-of-function phenotype in TNF-α-treated NPCs (Fig. 7e-g). In contrast, depletion of PUM1/2 using AAV in human NPCs abolished the NORAD-deficient phenotype in senescent NPCs (Fig. 7h, i). Furthermore, a surgically induced IVDD model was established by needle puncture in NORAD KO mice, and AAVs containing shPUM1/2 were used to target PUM1/2^13^. Histological analysis showed, according to morphological change analysis of IVDs, contrast with control surgical NORAD KO group, blockade of PUM1/2 in KO mice could restore the degeneration of IVD induced by a puncture to some extent (Fig. 7j, k). Radiographic results of intervertebral disc hight (IDH) assessment and histological score further confirmed this conclusion (Fig. 7l). The above data established that NORAD deficiency contributes to the aging of NPCs by negatively regulating PUMILIO activity.

### PUMILIO drives NPC senescence by repressing E2F3

**Fig. 8:**
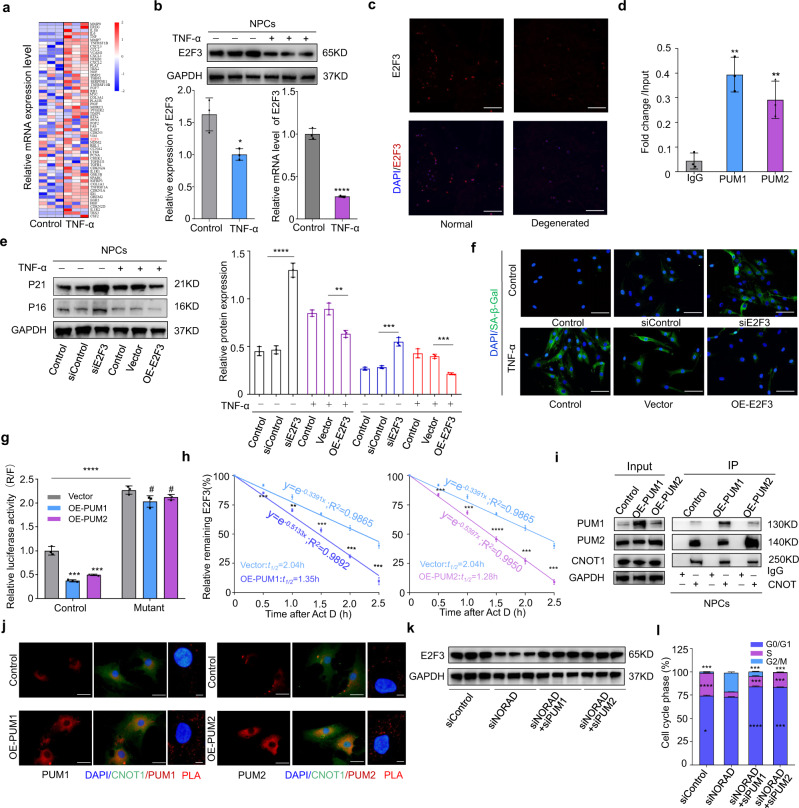
PUMILIO drives NPC senescence by repressing E2F3. **a** Heatmap of senescence-associated genes in NPCs by sequencing. **b** Expression of E2F3 in NPCs by western blot (*P* = 0.0167) and RT-qPCR (*P* < 0.0001). Data are shown as the mean ± SD, *n* = 3, *P < 0.05, ****P < 0.0001, two-tailed unpaired Student’s *t* test. **c** IF of E2F3 in NP tissues (red: E2F3; blue: DAPI; scale bar: 200 μm). **d** RIP-qPCR and RNA pulldown assay to determine the binding status of PUM1 (*P* = 0.0014) and PUM2 (*P* = 0.0064) with E2F3 transcripts. Data are shown as the mean ± SD, *n* = 3, **P < 0.01, two-tailed unpaired Student’s *t* test. **e** Protein level analysis of P21 and P16 in normal (*P* < 0.0001; *P* = 0.0034) and senescent NPCs (*P* = 0.0006; *P* = 0.0002) with or without E2F3 silencing or overexpression, GAPDH was used as the loading control. Data are shown as the mean ± SD, *n* = 3, **P < 0.01, ***P < 0.001, ****P < 0.01, two-tailed unpaired Student’s *t* test. **f** IF analysis of SA-β-gal activity (green: SA-β-gal; blue: DAPI; scale bar: 100 μm) in NPCs with E2F3 silencing (siE2F3) or overexpression (OE-E2F3). **g** Dual-luciferase reporter assay of NPCs containing control (*P* = 0.0003; *P* = 0.0006) or mutPRE (*P* = 0.0521; *P* = 0.0703) with or without PUM1/2 overexpression. Data are shown as the mean ± SD, *n* = 3, ^#^NS, ***P < 0.001, ****P < 0.0001, two-tailed unpaired Student’s *t* test. **h** Stability of E2F3 transcript in senescent NPCs with PUM1 (*P* = 0.0006; *P* = 0.001; *P* = 0.0002; *P* = 0.001; *P* = 0.001) or PUM2 (*P* = 0.0008; *P* = 0.0008; *P* < 0.0001; *P* = 0.0001; *P* = 0.0001) overexpression. Data are shown as the mean ± SD, *n* = 3, **P < 0.01, ***P < 0.001, two-tailed unpaired Student’s *t* test. **i** Co-IP analysis of CNOT1 interaction with PUM1 or PUM2 in NPCs with or without PUM1/2 overexpression. **j** IF analysis of the interaction of CNOT1 with PUM1 or PUM2 in NPCs with or without PUM1/2 overexpression (red: PUM1/2; green: CNOT1; blue: DAPI; scale bar: 50 μm); Interaction of CNOT1 with PUM1 or PUM2 by PLA (red: Signal; blue: DAPI; scale bar:10 μm). **k** Expression of E2F3 in NORAD-silenced NPCs with or without PUM1/2 silencing. **l** Cell cycle analysis of NORAD-silenced NPCs with or without PUM1/2 silencing at G0/G1 (*P* = 0.0125; *P* < 0.0001; *P* < 0.0001), S (*P* < 0.0001; *P* < 0.0001; *P* < 0.0001), G2/M phase (*P* < 0.0001; *P* < 0.0001; *P* < 0.0001). Data are shown as the mean ± SD, *n* = 3, *P < 0.05, **P < 0.01, two-tailed unpaired Student’s *t* test.

The Pumilio proteins PUM1 and PUM2 are members of the PUF family of sequence-specific RNA-binding proteins, which bind an extensive network of mRNAs and regulate diverse cellular processes by promoting mRNA decay^41^. We hypothesize that PUM1 and PUM2 promote cellular senescence by promoting the decay of target mRNAs. To further elucidate the specific mechanism, we analyzed the genes involved in cellular proliferation and the aging process in NPCs and found that E2F3, a target of PUM1/2, was also among the most significantly downregulated genes after treatment with TNF-α, which was further confirmed by western blot and RT-qPCR analysis (Fig. 8a, b). Furthermore, fluorescence immunostaining of E2F3 confirmed the decreased expression of E2F3 in degenerated NP tissues (Fig. 8c). To assess whether E2F3 is regulated by PUM1/2 at the posttranscriptional level, RIP-qPCR was performed to determine the binding status of PUM1/2 with E2F3 transcripts (Fig. 8d). Knockdown of E2F3 in NPCs inhibited cell proliferation and promoted cellular senescence, while overexpression of E2F3 partially blocked the pro-senescence effect of TNF-α in NPCs (Fig. 8e, f). The surgically induced IVDD model using AVV containing shE2F3 and OE-E2F3 confirmed these results in vivo (supplementary Fig. 6a–c). The interaction of Pumilio proteins with mRNAs depends on the presence of a canonical UGUARAUA sequence in the mRNA 3’UTR named the Pumilio response element (PRE), and two PRE sites are present in the 3’UTR of E2F3 (supplementary Fig. 6d). Dual-luciferase reporter plasmids containing the E2F3 3’UTR carrying the wild-type PRE or a mutant PRE in which the 5-UGUACAUA-3 motif (wtPRE) had been mutated to 5-ACAACATA-3 (mutPRE) were transfected into NPCs with PUM1/2 overexpression plasmids. The luciferase activities in NPCs expressing mutated PREs were significantly higher than those in wild-type NPCs, and the overexpression of PUM1 or PUM2 had little influence on mutPRE-containing E2F3, further confirming the binding region and binding sites of PUM1/2 (Fig. 8g). Furthermore, mRNA stability assays revealed that overexpression of PUM1 or PUM2 in NPCs decreased the half-life of E2F3 transcripts (Fig. 8h), a finding which was validated by the increased interaction between PUM1 or PUM2 and CNOT1 shown by co-immunoprecipitation, immunofluorescence, and proximity ligation assays (Fig. 8i, j and Supplementary Fig. 6e). In addition, knockdown of NORAD downregulated the expression of E2F3 in NPCs and reduced the S-phase population, which were restored by the additional depletion of PUM1/2 (Fig. 8k, l). Collectively, these data showed that PUMILIO protein drives NPC senescence by repressing E2F3 at the posttranscriptional level.

## Discussion

**Fig. 9:**
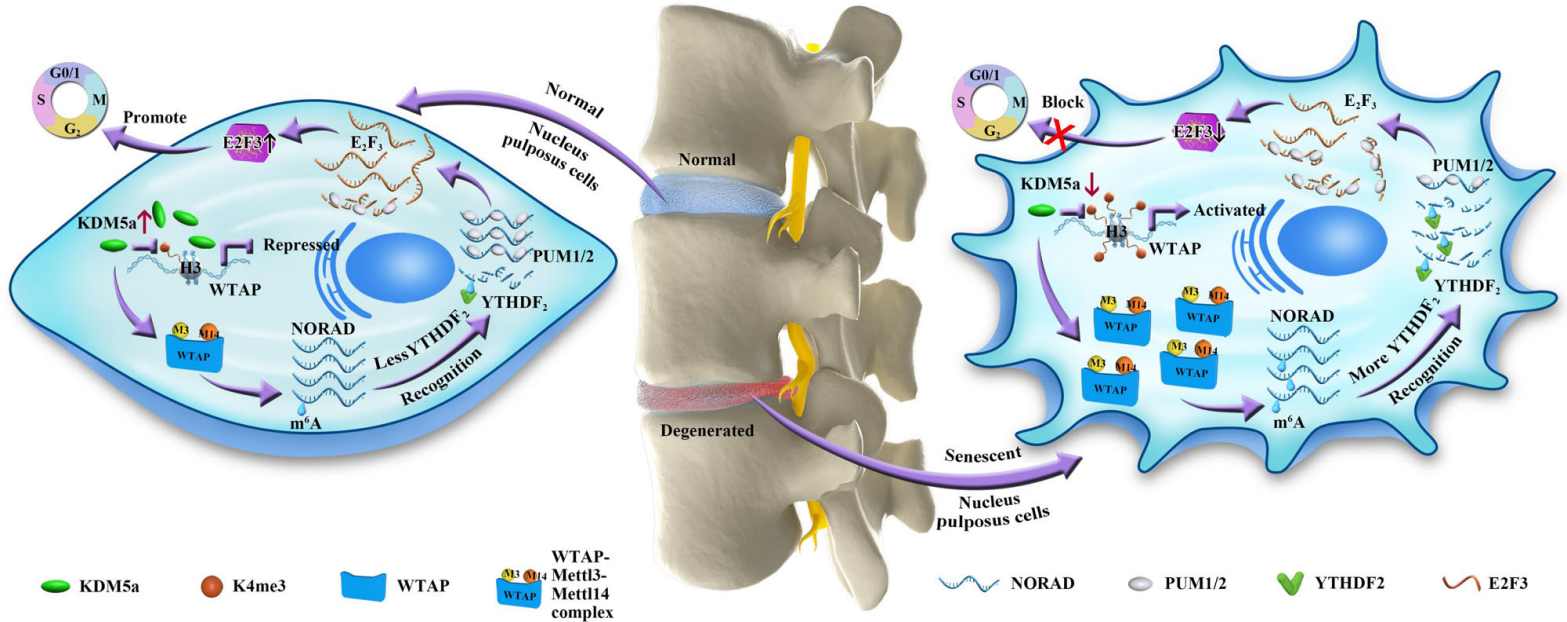
Schematic representation of the mechanisms via which m^6^A modification mediates NPC senescence and IVDD progression. In senescent NPCs, downregulated KDM5a enhances H3K4me3 modification of the WTAP promoter and promotes WTAP expression. WTAP promotes m^6^A modification of NORAD and induces degradation of NORAD transcripts by YTHDF2 recognition. Less NORAD results in less sequestered RNA-binding proteins PUM1/2, leading to more binding of PUM1/2 to E2F3 mRNAs. This leads to more recognition of PUM1/2 and degradation of E2F3 transcripts, promoting the senescence of NPCs.

The pathogenesis of IVDD is a complex aging-associated process to which NPC aging contributes greatly^32,42^. Recent studies have revealed that m^6^A modification regulates multiple musculoskeletal disorders^43–46^. In this study, we first revealed that the lncRNA NORAD could be modified by m^6^A due to an increase in WTAP, which was regulated by KDM5a-mediated H3K4me3 modification of the promoter. NORAD was downregulated during disc degeneration and NPC senescence, while overexpression of NORAD inhibited NPC senescence in vitro. Further investigation found that fewer of the RNA-binding proteins PUM1/2 were sequestered when NORAD was downregulated, in turn increasing the degradation of targeted mRNAs of E2F3, thus promoting the senescence of NPCs (Fig. 9).

The m^6^A modification is regulated by a methyltransferase complex and demethylases^47^. WTAP binds METTL3 and METTL14, forming the methyltransferase complex which is responsible for m^6^A modification, while FTO and ALKBH5 function as demethylases, catalyzing the reverse effect. Previous studies have shown that m^6^A modification can alleviate the aging process. Premature aging of human MSCs (mesenchymal stem cells) can be mitigated by methylation of MIS12 mediated by elevated expression of METTL3. Targeting METTL3 reduces m^6^A-modified MIS2, resulting in decline in MIS12 mRNA stability, less IGF2BP2 recognition, thus accelerating the senescence of hMSCs^48^. In addition, a recent report revealed that m^6^A modification of the cell cycle regulator PLK1 rescued the senescence and apoptosis of dental pulp stem cells by regulating the cell cycle^49^. The senescence of NPCs contributes a lot to the degeneration of human intervertebral discs. However, little is known about the regulation of NPC senescence at the epitranscriptional level.

In this study, we focused on the regulation of m^6^A modification in the senescence of NPCs and found that WTAP was obviously upregulated and interacted with METTL3 and METTL14 to increase the formation of methyltransferase complexes in senescent NPCs. Recent researches have shown that ALKBH5 and METTL3 can be regulated by epigenetic modification of their promoters in tumor progression^19,50^. Further m^6^A modification regulates histone modification and crosstalk with H3K27me3 through decay of KDM6B mRNA in the innate immune response^51^. Histone modification could serve as a key regulatory method of inducing epigenetic alterations during the aging process^52^. In addition, increased modifications of H4K16ac and H3K4me3, as well as decreased H3K9me3 and H3K27me3 constitute the epigenetic signature of senescence^36^. We next investigated whether the upregulation of WTAP in NPCs was mediated by histone modification, and found that KDM5a-mediated H3K4me3 modification of the WTAP promoter facilitates its transcription, thus promoting m^6^A modification of the lncRNA NORAD. The fate of transcripts harboring m^6^A was determined by recognized readers. To clarify the binding reader of m^6^A-modified NORAD, we performed RNA-pulldown and CLIP assays and confirmed that YTHDF2 recognized methylated NORAD as a reader, resulting in its decreased stability and expression.

NORAD is a lncRNA that is regulated in response to DNA damage and plays a key role in maintaining genome integrity^40,53^. A previous study showed that NORAD sequestered two PUMILIO proteins (PUM1 and PUM2) that regulate a variety of targets during cell growth and aging in a lncRNA-RBP regulatory interaction mode. PUM1/2 are RNA-binding proteins that act as negative regulators of gene expression by binding 3’-UTRs of target transcripts. In this study, we confirmed by RNA pull-down and RIP-qPCR that NORAD sequestered PUM1/2 and inhibited their pro-decay function in NPCs, which was attenuated in senescent NPCs due to the increase in m^6^A-mediated decay of NORAD. We further found, in senescent NPCs, that PUM1/2 targeted and degraded more transcripts of E2F3, a key regulator of the cell cycle and proliferation, thus contributing to NPC senescence. However, whether other target transcripts of PUM1/2 mediate the critical function of the NORAD-PUM1/2 axis in the senescence of NPCs and pathogenesis of IVDD remains unclear and further investigations are needed to reveal broader insights during this process.

In summary, our research puts forward a mechanism in the process of NPC senescence mediated by m^6^A regulation, revealing that interruption of NORAD m^6^A modification or targeting of the NORAD/PUMILIO/E2F3 axis could alleviate the progression of IVDD, and further provides a potential epigenetic therapeutic strategy to treat IVDD.

## Methods

### Ethics approval and consent to participate

This study protocol of using patient samples was approved by the Ethics Committee of Tongji Medical College, Huazhong University of Science and Technology (No. S341). Protocol of animal model of intradiscal injection was approved by The Institutional Animal Care and Use Committee (IACUC) at Tongji Medical College, Huazhong University of Science and Technology (No. S2394). The study was performed in accordance with the Declaration of Helsinki.

### Patient samples

NP tissues were obtained from 68 patients (38 females and 30 males; age 54.2 ± 8.4 years) with degenerative disc disease undergoing surgery. The control samples were taken from 71 patients (42 females and 29 males; age 25.2 ± 14.2 years) undergoing surgery due to scoliosis or thoracolumbar fracture after informed consent was obtained. This study protocol was approved by the Ethics Committee of Tongji Medical College, Huazhong University of Science and Technology (No. S341).

### Me-RIP-seq

Six samples of NPCs with three replicates for each group were used to extract RNA using TRIzol™ Reagent (ThermoFisher, Massachusetts, USA). RNA fragmentation, m^6^A-IP, and library preparation were carried out according to previously published protocols^54^. RNA was extracted from NPCs with using a phenol-chloroform lysate to obtain a purified product. Then polyA-mRNA was enriched using Dynabeads™ mRNA DIRECT™ Purification Kit (ThermoFisher, Massachusetts, USA) according to the instruction. And RNA fragments were incubated with anti-N6-methyladenosine (m^6^A) antibodies (Sigma-Aldrich, Burlington, USA) in immunoprecipitation (IP) buffer for 2 h at 4 °C. The samples were then washed with low-salt precipitation buffer and high-salt buffer thrice continuously. For library construction, the purified RNA was collected for the generation of an RNA-seq library with NEBNext R Ultra™ RNA Library Prep Kit (New England Biolabs, MA, USA). The quality of the library was done using Bioptic Qsep100 analyzer and sequencing was done using NovaSeq’s high-throughput sequencing platform. 150 bp paired-end reads were mapped to the reference human genome build GRCh38/hg38 by using Bowtie2. Only unique mapping paired reads were sorted using SAMtools and kept for subsequent analysis. Peaks of each sample and differential methylation peak were called using the exomePeak R-package. GRCh38/hg38 was used for human genome build and peak files were generated using the exomePeak R package. For Me-RIP-seq data analysis, m^6^A IP was normalized to input, and m^6^A peaks were identified using exomePeak R package (v2.13.2) under parameters: “PEAK_CUTOFF_PVALUE = 0.05,PEAK_CUTOFF _FDR = NA,FRAGMENT_LENGTH = 200”.

### Single-molecule FISH (fluorescence in situ hybridization)

Single-molecule FISH was performed according to the RNA scope Fluorescent Multiplex kit instructions (Advanced Cell Diagnostics, Hayward, CA, USA). RNA scope technology allows for the detection of single RNA molecules by way of its zz oligo pair design and DNA-based amplification methods^55^. The NORAD probe was designed against nucleotides 3352-4908. Slides were washed with 10% formamide in 2× SSC and stained with DAPI. Then images were captured under a microscope (Olympus, BX53; Melville, NY, USA).

### IF staining of β-galactosidase staining in cells

Cells were incubated with 33 mM β-galactosidase substrate C_12_FDG (fluorescein di-B-d-galactopyranose) in 2 mL medium for 2 h, after pretreatment with 100 nM bafilomycin A1 for 1 h at 37 °C. After incubation, the cells were washed with PBS and fixed with 4% paraformaldehyde for 15 min at room temperature. Then nuclei were counter-stained using 0.1 g/mL DAPI (Beyotime, Shanghai, China). This was followed by visualization and capturing of the images under a microscope (Olympus, BX53). The experiments were replicated three times.

### RNA pull-down assay

Fragments (704-1322) of NORAD and NORAD-AS were amplified with primers containing T7 and SP6 promoter sequences (NORAD:*TAATACGACTCACTATAGGGAGA*CCACCCTC TGGGAAGATTTACTG; NORAD-AS: *ATTTAGGTGACACTATAGAAGGG*AACAGGTGATTTGGCCATTCCCC). The templates were transcribed using MAXIscript™ SP6/T7 Transcription Kit in vitro (ThermoFisher Scientific, MA, USA). And then Pierce™ RNA 3′ End Desthiobiotinylation Kit (ThermoFisher Scientific, MA, USA) was applied to obtain biotin-labeled NORAD or NORAD-AS. After treatment with DNase I and the RNeasy kit (Qiagen), In vitro transcribed RNA was treated. 40 pmol of purified RNA in 60 µL RNA structure buffer (10 µM Tris-Cl pH 7.0, 0.1 M KCl, 10 mM MgCl_2_) was heated to 95 °C for 5 min, then put on ice for 3 min, and left at room temperature for 20 min to allow proper secondary structure formation. In total, 3 × 10^7^ NPCs lysis were freshly prepared with Anti-RNase, Protease/Phosphatase Inhibitor Cocktail supplemented in 1.3 mL lysis buffer [150 mM NaCl, 50 mM Tris-Cl, pH 7.5, 0.5% Triton X-100, 1 mM PMSF]. Lysates were then sonicated using a Bioruptor (Diagenode) for 10 min with 30 s on/off cycles and pre-cleared with 50 µL washed Dynabeads^®^ MyOne^TM^ Streptavidin C1 (Invitrogen, 65002) at 25 for 3 h. And 40 pmol folded RNA were incubated with 10 mg cell lysates and rotated at 4 °C for 2 h, followed by the addition of 50 µL streptavidin C1 Dynabeads and further rotation for 1 h. Beads were washed 6 times with lysis buffer at 4 °C and proteins were eluted by incubating in RNase A buffer (50 mM Tris-Cl pH 7.5, 150 mM NaCl, 100 µg/mL RNase A) for 35 min at 37 °C. Then eluted proteins from samples were collected and subjected for western blot.

### Subcellular fractionation for RT-qPCR

RNA extraction was performed using an RNA subcellular isolation kit (Active Motif Catalogue No. 25501) and real-time PCR was performed according to the protocol used in our previous study^56^. GAPDH was used as the endogenous control for cytoplasmic RNA, while 18S RNA was selected as the endogenous control for nuclear RNA.

### RNA stability assay

After treatment with 5 μg/ml actinomycin D (MedChem Express) to inhibit mRNA transcription, cells were collected at 0, 3, and 6 h to analyze mRNA levels and the rate of degradation. Total RNA was extracted and used for RT-qPCR. The degradation rate of RNA (k) was calculated using the equation: e^-*kt*^ = N_0_/N_*t*_, where *t* means the time after transcription inhibition, *k* represents the degradation rate, and N_*t*_ and N_0_ are the relative mRNA expression levels at time *t* and time 0. The RNA half-lifetime(*t*_1/2_) was calculated from the degradation rate as *t*_1/2_ = ln2/k.

### Statistical analysis and reproducibility

Data are presented as the mean ± SD of at least three independent experiments. Statistical analyses were performed using Graphpad Prism 8.0.1 (GraphPad Inc., La Jolla, CA, USA). Two-tailed unpaired Student’s *t* test and two-way ANOVA followed by the Tukey–Kramer test were used to assess the statistical significance of any differences. *P* < 0.05 was considered statistically significant while *P* > 0.05 was considered nonsignificant (ns) (^*#*^*P* > 0.05, *P < 0.05, **P < 0.01, ***P < 0.001, and ****P < 0.0001). At least three independent experiments were repeated with similar results.

More supplementary Materials and Methods are available in Supplementary Information.

### Reporting summary

Further information on research design is available in the Nature Research Reporting Summary linked to this article.

## Supplementary information

Supplementary Information

Reporting Summary

## Data Availability

The raw data from the Me-RIP-Seq analysis of NPCs have been deposited in the Gene Expression Omnibus database under the accession code GSE169484. The NGS data of single NPCs in this study are available under the accession code GSE167931. The data supporting the findings of this study are available from the corresponding authors upon reasonable request.  Source data are provided with this paper.

## Source data

Source Data
