# Author Correction: WTAP-mediated m^6^A modification of lncRNA NORAD promotes intervertebral disc degeneration

**DOI:** 10.1038/s41467-022-31302-7

**Published:** 2022-06-22

**Authors:** Gaocai Li, Liang Ma, Shujie He, Rongjin Luo, Bingjin Wang, Weifeng Zhang, Yu Song, Zhiwei Liao, Wencan Ke, Qian Xiang, Xiaobo Feng, Xinghuo Wu, Yukun Zhang, Kun Wang, Cao Yang

**Affiliations:** 1 Department of Orthopaedics, Union Hospital, Tongji Medical College, Huazhong University of Science and Technology, 430022 Wuhan, China; 2 Department of Cardiology, Union Hospital, and Key Laboratory of Biological Targeted Therapy of the Ministry of Education, Tongji Medical College, Huazhong University of Science and Technology, 430022 Wuhan, China

**Keywords:** Senescence, Epigenetics, Mechanisms of disease, Long non-coding RNAs, RNA modification

Correction to: *Nature Communications* 10.1038/s41467-022-28990-6, published online 18 March 2022.

The original version of this Article contained an error in Fig. 6a, in which the western blot image of IGF2BP1 was mistakenly reproduced for IGF2BP3. This has been corrected in both the PDF and HTML versions of the Article.

