# Supplementary material for: WTAP-mediated m6A modification of lncRNA NORAD promotes intervertebral disc degeneration
Source: Nat Commun. 2022 Mar 18;13:1469. doi: 10.1038/s41467-022-28990-6 (PMC8933458; doi:10.1038/s41467-022-28990-6)
Supplement: Supplementary file 1 — Supplementary Information [file 41467_2022_28990_MOESM1_ESM.pdf]

Supplementary Information File

WTAP-mediated m^{6}A modification of lncRNA NORAD promotes intervertebral disc degeneration

Gaocai Li[1], Liang Ma[1], Shujie He[2], Rongjin Luo[1], Bingjin Wang[1], Weifeng Zhang[1], Yu Song[1], Zhiwei Liao[1], Wencan Ke[1], Qian Xiang[1], Xiaobo Feng[1], Xinghuo Wu[1], Yukun Zhang[1], Kun Wang[1*], Cao Yang[1*]

These authors contributed equally: Gaocai Li, Liang Ma, Shujie He, Rongjin Luo

[*]**Corresponding Authors**: Kun Wang; Cao Yang

E-mail: kunwangunion@hust.edu.cn (Kun Wang); caoyangunion@hust.edu.cn (Cao Yang)

This file includes:

Supplementary Materials and Methods

Supplementary Figures. 1 to 7

Supplementary Tables 1 to 4

Supplementary Materials and Methods

Generation of Norad KO mice

Norad KO mice were generated using CRISPR/Cas9-mediated genome editing technology by Cyagen (Guangzhou, China). The gRNA to mouse Norad, and Cas9 mRNA were co-injected into fertilized mouse eggs to generate targeted knockout offspring. F0 founder animals were identified by PCR followed by sequence analysis, which were bred to wildtype mice to test germline transmission and F1 animal generation. PCR screening and sequencing were performed to confirm the knockout effect. Then, inter-cross heterozygous targeted mice to generate homozygous targeted mice.

RNA-seq on isolated NPCs

Nine samples of NPCs with four replicates for normal group and five replicates for degenerated group were used for sequencing. Cells are centrifuged and RNA was harvested using TRIzol™ Reagent (Thermofisher, Massachusetts, USA). Illumina TruSeq RNA Sample Prep Kit (Cat#FC-122-1001) was used with 1 ug of total RNA for the construction of sequencing libraries. Libraries were prepared according to instructions of Illumina HiSeq X ten accompanying the Ovation RNA-Seq System V2 (M01206 v9). Briefly, RNA was elutated according to the manuscription user guide. Then RNA was fragmented using fragment buffer. cDNA was synthed using dNTPs, DNA polymeraseI and RNaseH. Then cDNA was purified using AMPure XP beads. PCR Was used to amplify with Illumina primers for 15 cycles and library fragments of ~200bp (insert plus adaptor and PCR primer sequences) were band isolated from an agarose gel. The purified RNA was captured on an Illumina flow cell for cluster generation. One normal sample and two degenerated samples were abandoned for

unquatification for quality control and three samples in normal and degenerated groups were performed following analysis. Libraries were sequenced on the Illumina HiSeq X ten following the manufacturer's protocols.

Illumina Casava1.7 software used for basecalling. Sequenced reads were qualified using FastQC and masked for low-complexity or low-quality sequence, then mapped to Homo_sapiens.GRCh38.94 whole genome. Reads Per Kilobase of exon per Megabase of library size (RPKM) were calculated using a protocol from Feng, Jianxing et al. Bioinformatics (Oxford, England) vol. 28,21 (2012): 2782-8. In short, exons from all isoforms of a gene were merged to create one meta-transcript. The number of reads falling in the exons of this meta-transcript were counted and normalized by the size of the meta-transcript and by the size of the library. Homo_sapiens.GRCh38.94 was used for human genome building. For RNA-seq data analysis, differential gene expression analysis was performed using the DESeq2 R-package, and the criteria of diff genes was "fold change >= 2, FDR < 0.05". Principal component analysis was achieved using Stats R package (3.6.1).

Gene set enrichment analysis (GSEA)

GSEA was performed using GSEA software version 3.0 with 1,000 permutations and default parameters according to standard procedure (http://www.broadinstitute.org/gsea/doc/GSEAUserGuideFrame.html) as described by GSEA user guide. Differential gene expression analysis was performed using the DESeq2 R-package, and the criteria of diff genes was "FC >= 2, FDR < 0.05". The curated gene set C2 of the Molecular Signature Database version 4.0 was used to compute overlaps between gene sets in Molecular Signature Database and our gene set.[1]

Nucleus pulposus cells isolation and culture

The NP tissue samples were separated, cut into pieces, and treated with 0.2% collagenase type II (Invitrogen, Carlsbad, CA, USA) for 8 h at 37 ℃. The digest was centrifuged at 1200 rpm and then cultured in Dulbecco's Modified Eagle's Medium (DMEM; Gibco, Grand Island, NY, USA) with 10% fetal bovine serum (FBS; Invitrogen), 1% penicillin–streptomycin (Sigma), 2 mM glutamine (Sigma), and 50 μg/mL L-ascorbic acid (Sigma) at 37℃ in 5% CO_2. When grew to confluence, the cells were digested by 0.25% trypsin/1 mM EDTA and passed for expansion. The NPCs from passage 2 were plated into experimental plates for following experiments. In some experiments, cells were stimulated by 20ng/ml of TNF-α (Peprotech, 300-01A, ROCKY HILL, New Jersey) in the culture medium for 24 h. Unstimulated cells were used as controls.

Western blot

Samples were lysed by RIPA (Beyotime, P0013B, Shanghai, China) and their protein contents were measured using the Micro BCA Protein Assay Kit (Beyotime, P0010S, Shanghai, China). SDS-polyacrylamide gel electrophoresis gels were used for electrophoresis and then transferred to PVDF membranes. The membranes were incubated with the specific primary antibody (The primary antibodies used were listed in ***Supplementary Table 1***) overnight at 4 °C after blocking with Blocking buffer and HRP-conjugated Affinipure Goat Anti-Rabbit IgG (SA00001-2, Proteintech,1:10000) and HRP-conjugated Affinipure Goat Anti-Mouse IgG (SA00001-1, Proteintech, 1:1000) were added. Protein expression was visualized using enhanced chemiluminescence reagents (Affinity, KF001, Nanjing, China) and the ChemiDoc MP Imaging System (Bio-Rad, 12003154 Hercules, CA, USA). And Image J (V1.52v)was used

for semi-quantification of the expression of proteins.

Me-RIP-PCR

A previously described procedure was used for methylation RIP (Me-RIP)[2]. The Dynabeads mRNA Purification Kit (Invitrogen) was used to purify mRNA from the total RNA. Magna MeRIP™ m^6A Kit (Millipore) was used to measure the change of m^6A levels in mRNA according to the manufacturer's protocol after RNA quality analyzing by NanoDrop 2000. After saving 0.5μg of the mRNA as input, the remaining mRNA was used for m6A immunoprecipitation. After immunoprecipitation with Magna ChIP protein A/G Magnetic Beads and eluted twice with elution buffer, immunoprecipitated m6A RNAs were recovered by ethanol precipitation, and the RNA concentration was measured with NanoDrop 2000. Then immunoprecipitated m^6A RNA was used as templates in RT-qPCR, as described above. Bio-rad CFX Connect Real-Time Systerm was used for PCR and Image Lab (6.1) was used for data analysis. The primers used were listed in supplementary ***Supplementary Table 3***. The housekeeping gene HPRT1 was chosen as internal control since HPRT1 mRNA dose not have m^6A peaks in m^6A profiling.

NPC transcript sequencing

Nucleus pulposus cells treated with TNF-α or not were used for next-generation transcript sequencing. For RNA-seq data analysis, differential gene expression analysis was performed using the DESeq2 R-package, and the criteria of diff genes was "fold change >= 2, FDR < 0.05". The different expression of gene transcripts was analyzed and visualized using R heatmap and stats Package.

Immunohistochemistry (IHC)

For IHC, paraffin-embedded tissue sections were deparaffinized with xylene and rehydrated with an alcohol gradient and water. Sections were incubated with primary antibodies P16 (diluted 1:500, CST, catalogue number: #80772), P21 (diluted 1:100, CST, catalogue number: #2947), MMP13 (diluted 1:100, Abcam, catalogue number: ab39012) at room temperature for 1h and biotin-labelled secondary antibodies for 30min, and then stained with Vectastain ABC kit and DAB peroxidase substrate kit (Boster, AR1000).

Immunofluorescence staining

Immunofluorescence analysis was performed as following described. First, 4% paraformaldehyde was used to fix NPCs, and then 0.5% Triton X-100 in PBS was used to permeabilize. The slides were washed in PBS and blocked with 2% bovine serum albumin (BSA) in PBS for 2 h at 37 °C, and then incubated with anti-53 (1:800, #2527, CST), PUM1 (1:500, NB100-259, Novus), PUM2 (1:1000, ab92390, Abcam), Ki67 (1:400, #9449, CST), CNOT1 (1:50, 14276-1-AP, Peprotech) for 10 h. After washing twice, the slides were then incubated with goat anti-rabbit antibody (CST) at 37 °C for 1 h. Nuclei were then co-stained with 0.1 g/ml DAPI (Beyotime, Nantong, China), and images were captured under a microscope (Olympus, BX53; Melville,NY, USA).

SA-β-galactosidase staining.

Cells were first fixed in 2% formaldehyde and 0.2% glutaraldehyde at room temperature for 5 min and then stained using fresh staining solution at 37°C for 12 hours. SA-β-gal-positive cells were counted using flow cytometry (BD FACS Calibur; BD Biosciences, San Jose, CA, USA) and images captured under a microscope (Olympus, BX53; Melville, NY, USA).

Immunoprecipitation studies and western blot analyses.

Control cells or cells transfected with expression plasmids were lysed in HEPES lysis buffer after corresponding disposal (180 mM NaCl, 1.5 mM $MgCl_2$, 5 mM EDTA, 50 mM HEPES, 1% NP40, 10% glycerol, 0.1% sodium orthovanadate, and a mixture of protease inhibitors from Roche Applied Science). Lysates were immunoprecipitated (IP) with beads conjugated with antibodies. The associated proteins were separated on SDS–PAGE and then incubated with the specific primary antibody and visualized using enhanced chemiluminescence reagents (Affinity, KF001, Nanjing, China) and the ChemiDoc MP Imaging System (Bio-Rad, 12003154 Hercules, CA, USA).

RT-qPCR.

TRIzol reagent (Invitrogen) was used to extract total RNA from the cultured cells or clinical NP tissues. The extracted RNA was reverse-transcribed using cDNA Synthesis Kit (Vazyme, NanJing, R312-01) according to the manufacturer's instructions followed by RT-qPCR using RT SuperMix for qPCR (Vazyme, NanJing, R323-01). The primers used for RT-qPCR were listed in ***Supplementary Table 2***.

Chromatin Immunoprecipitation assays (ChIP).

Cells were conducted with corresponding disposal. ChIP was conducted using a Simple ChIP Enzymatic Chromatin IP kit (Cell Signaling Technology), according to the manufacturer's instructions. Antibodies of anti-H3K27me3, anti-H3K4me3, anti-H3K9me3, anti-H3K16ac, anti-KDM5a were used for immunoprecipitation. Normal rabbit IgG was used as a negative control. Primers of WTAP for ChIP-qPCR are listed in ***Supplementary Table 3***.

RNA interference and plasmid transfection

Knockdown of WTAP, KDM5a, YTHDF2, NORAD, PUM1, PUM2 and E2F3 in NPCs was realized by transfection with siRNA. siRNA against WTAP (siWTAP), KDM5a (si KDM5a), YTHDF2 (siYTHDF2), NORAD (siNORAD), PUM1 (siPUM1), PUM2 (siPUM2), E2F3

(siE2F3), and scrambled siRNA (siControl) were synthesized by Sangon Biotech (Shanghai, China) and transfected with Lipofectamine 2000 (Invitrogen) according to the standard protocol. The siRNA sequences were listed in *Supplementary Table 4*. After verified high silencing efficiency, the NP cells were then used in the following treatment. The psiCHECKTM-2 NORAD, pGL3-Basic-NORAD and pRL-TK plasmids using for luciferase analysis were constructed by TsingKe Biological Technology (Beijing, China) and pGL3-KDM5a, pGL3-PUM1, pGL3-PUM2, pGL3-E2F3 plasmids were constructed followed by lentivirus package by GenePharma (Shanghai, China) for overexpression experiments.

Proximity Ligation Assay.

Nucleus pulposus cells were fixed for treated with the indicated doses and times then fixed for 15 minutes by shaking at room temperature in 4% paraformaldehyde. After washing using PBS and permeabilization using 0.5% Triton. Then the Duolink® In situ PLA ® kit (SigmaAldrich) in mouse/rabbit (with red detection) was used according to the manufacturer's protocol (DUO92101-1kit). Antibody concentrations were optimized prior to PLA experiments, WTAP (Novus, NBP1-83040); METTL3(Abcam, ab195352); METTL14(Abcam, ab223090); CNOT1(Proteintech, 66507-1-Ig); PUM1(Abcam, ab92390); PUM2(Abcam, ab92390). Cells were mounted using Duolink® In Situ Mounting Medium (with DAPI) and cured, and sealed with clear nail polish prior to imaging. Images were taken on a Olympus standard inverted fluorescent microscope. The number of PLA signals per cell were quantified for three separate experiments. The statistical significance was determined using GraphPad Prism and shown as SEM in the figure. P values were determined using an unpaired student's t test.

Luciferase reporter assay

NP cells were seeded in triplicate in 24-well plates to allow 70% confluency in the next day. 100 ng reporter plasmids with NORAD exon region (psiCHECKTM-2 NORAD) or plasmids with promoter of NORAD (pGL3-Basic- NORAD) and vector 20 ng renilla luciferase (Rluc) control plasmids (pRL-TK) were co-transfected with or without WTAP knockdown using Fugene HD (Promega #E231A). Fluc and Rluc activities were measured 24-h later with the Dual-Luciferase Reporter Assay System (Promega) according to the instructions. The relative luciferase activity was calculated by dividing Fluc by Rluc and normalized to individual control for each assay.

Nuclear run-on assay

The nuclear run-on assay was conducted following the published protocol[3]. Cells were immediately washed once with ice-cold PBS after disposal, treated with trypsin-EDTA, pelleted by centrifugation at 400g for 4 min at 4°C, and lysed on ice for 5 min by NP-40 lysis buffer (10 mM tris-HCl, pH 7.4, 10 mM NaCl, 3 mM MgCl2, and 0.5% NP-40). The nuclei were collected and washed once with the lysis buffer. For nuclear run-on transcription, nuclei were resuspended in the transcription buffer (10 mM tris-HCl, pH 8.3, 2.5 mM MgCl2, 150 mM KCl, and 2 mM DTT) plus 100 U of RNaseOUT, 0.5 mM Br-UTP (bromouridine 5′-triphosphate), 0.5 mM UTP, 1 mM ATP (adenosine 5′-triphosphate), 1 mM CTP (cytidine 5′-triphosphate), and 1 mM GTP (guanosine 5′-triphosphate) at 30°C for 30 min. The nuclear RNA was extracted using the MEGAclear™ Transcription Clean-Up Kit (Invitrogen, AM1908) according to the manufacturer's instructions, followed by the removal of genomic DNA contamination with the TURBO DNA-free™ Kit (Invitrogen, AM1907). Labeled nascent transcripts were purified by IP using protein G Dynabeads (Invitrogen) and anti-BrdU (5-

bromo-2′deoxyuridine) monoclonal antibody (Abcam, ab6326). qPCR was used to determine the transcript levels using primers for WTAP: (forward) 5’-GGAAAGGACGGGGA GTGTTAC-3’; (forward) 5’-GCATTCGACACTTCGCCATT-3’ after the RNA extraction.

DNase I Sensitivity Analysis

Nucleus of NP cells stimulated with TNF-α 24 h were pretreated with DNase I (0.1 U/μl, Sigma) at 37°C for 20 min and then stopped by EDTA (50mM/ml). Genome DNA was extracted and subjected to quantitative RT -PCR assay for detection of WTAP promoter region using ChIP primers.

Cell cycle analysis

The Cell Cycle and Apoptosis Analysis Kit (Beyotime, C1052, Shanghai, China) was used for the cell cycle analysis according to the manufacturer’s instructions. Analysis was then performed using flow cytometry (BD FACS Calibur; BD Biosciences, San Jose, CA, USA) and analyzed using flowjo V10 (BD biosciences, Franklin Lakes, New Jersey).

Animal model of intradiscal injection

Protocol was approved by The Institutional Animal Care and Use Committee (IACUC) at Tongji Medical College, Huazhong University of Science and Technology (NO. S2394). The surgical procedure was performed as described previously[4]. The mice were raised under SPF condition, with 12 hours of dark/light cycle, with 23°C ambient temperature and 50% humidity. After 50g male NORAD-KO or C57 mice at age of 8 weeks were anesthetized with 3% (w/v) pentobarbital (2ml/kg) and grouped randomly, investigators blinded to the group allocation performed the experiment. The disc levels in rat tail (Co6/7, 7/8, and 8/9) were located by palpation on the coccygeal vertebrae and confirmed by trial radiography. Needles (33-G) were

used to puncture the annulus fibrosus layer though the tail skin, in parallel to the end plates. To ensure that the needle did not penetrate too deeply, the length of the needle was pre-determined according to the dimensions of annulus fibrosus and the NP, which were measured in a preliminary experiment and found to be approximately 4 mm. Five kinds of solution were prepared for intradisc injection, including AAV vector, AAV containing shPUM1, AAV containing shPUM2 for Norad KO mice, AAV vector, AAV containing shE2F3, AAV containing OE-E2F3 for WT mice. Each segment was injected with 20μl of the solution of interest, and each needle were kept in the disc for 10s. All animals were allowed free, unrestricted weight bearing and activity. The injections were conducted each week for one month and then keep for one month.

Histological and radiographic evaluation and analysis

Two months later, mice were sacrificed and histological and radiographic evaluation were performed. After X-ray and MRI examination, tails were fixed in 10% neutral-buffered formalin for 1 week and Midsagittal sections were stained with hematoxylin and eosin and Safranin O-fast green. The histological evaluation was performed according to histologic grading system developed by Ji et al[4]. More specifically, the cellularity and morphology of the AF, NP, and the border between the two structures were examined. The scale is based on 5 categories of degenerative changes with scores ranging from 0 points (0 in each category) for a normal disc to 15 points (3 in each category) for a severely degenerated disc. For morphology of the NP, score 0: round shape and the NP constitutes >75% of the disc area, score 1: round shape and the NP constitutes 50–75% of the disc area, score 2: round shape and the NP constitutes 25–50% of the disc area, score 3: round shape and the NP constitutes <25% of the

disc area. For cellularity of the NP, score 0: stellar-shaped cells with a proteoglycan matrix located at the periphery, evenly distributed, score 1: partially stellar and partially round cells, more stellar than round, score 2: mostly large, round cells, separated by dense areas of proteoglycan matrix, score 3: large, round cells, separated by dense areas of proteoglycan matrix. For morphology of the AF, score 0: well-organized collagen lamellae with no ruptures, score 1: inward bulging, ruptured, or serpentinefibers constitute <25% of the AF, score 2: inward bulging, ruptured, or serpentinefibers constitute 25−50% of the AF, score 3: inward bulging, ruptured, or serpentinefibers constitute >50% of the AF. For cellularity of the AF, score 0: fibroblasts comprise >90% of the cells, score 1: fibroblasts comprise>75–90% of the cells, score 2: intermediate, score 3: chondrocytes comprise >75% of the cells. For border between the NP and AF, score 0: normal, without any interruption, score 1: minimal interruption, score 2: moderate interruption, score 3: severe interruption. Radiographs were taken at 6 and 12 weeks after the puncture. The change in IVD height was evaluated by the disc height index (DHI) The change in IVD height was evaluated by the disc height index (DHI) Measurements of internal control discs were carried out together with their corresponding punctured discs. Disc height and the adjacent vertebral body heights were measured on the midline and 25% of the disc's width from the midline on either side. The DHI was expressed as the mean of the 3 measurements from midline to the boundary of the central 50% of disc width divided by the mean of the 2 adjacent vertebral body heights. Disc height indexes (DHIs) were calculated by DHI=1/2 x IVD height/two adjacent IVD body height.

Supplementary Figures

Figure 1

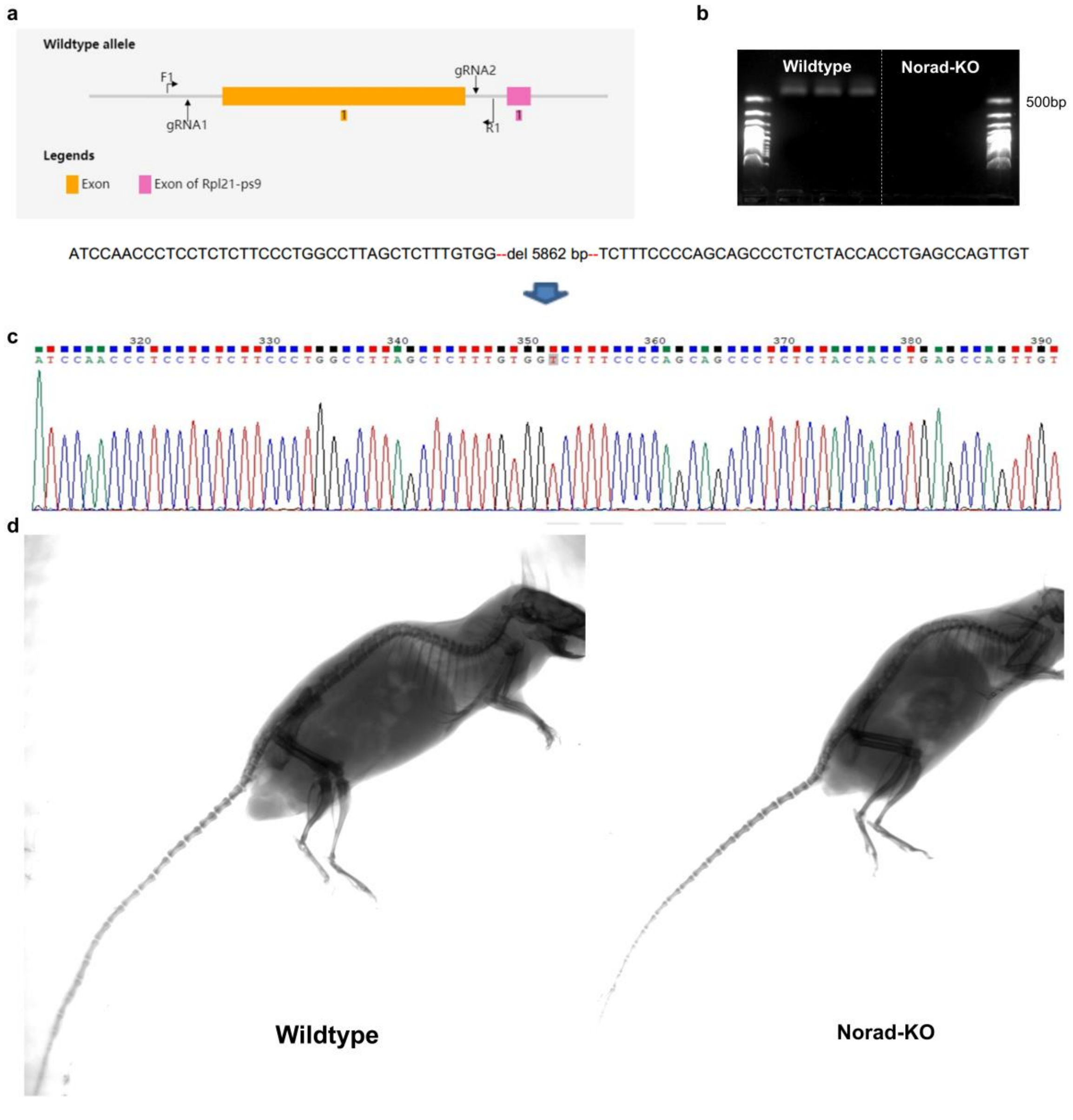

Supplementary Fig. 1 (**a**) Genotyping Strategy of NORAD KO mice using CRISPR/Cas9-mediated genome editing technology. (**b**) Genotyping using tail DNA of wild type (WT) and NORAD KO mice, three independent experiments were repeated. (**c**) Genotyping of KO mice was confirmed by Sequencing. **(d)** Representative x-ray images of 10-week-old WT and KO mice, manifesting kyphosis and narrowed disc space in KO mice.

Figure 2

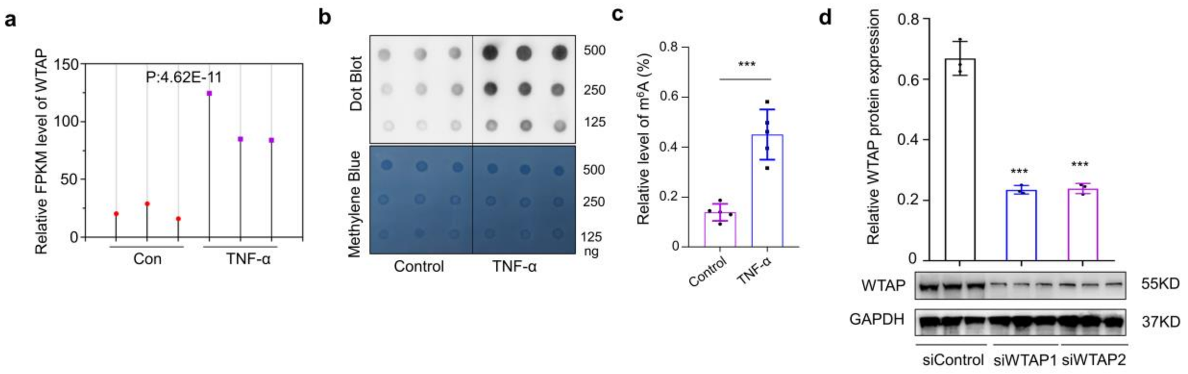

Supplementary Fig. 2 (a) FPKM level of WTAP transcripts in NPCs treated with TNF-α or not by NGS. **(b, c)** Overall m^6A modification in normal and senescent NPCs by Dot Blot and m^6A colorimetric assay; Methylene Blue was used as loading control. Data are shown as the mean ± SD from five independent experiments, p=0.0002, ***P<0.001, two-tailed unpaired Student's t-test. **(d)** Knockdown efficiency of WTAP in human NPCs using two specific siRNAs confirmed by western blot and RT-qPCR. Data are shown as the mean ± SD from three independent experiments, p=0.0002, p=0.0002; ***P<0.001, two-tailed unpaired Student's t-test.

Figure 3

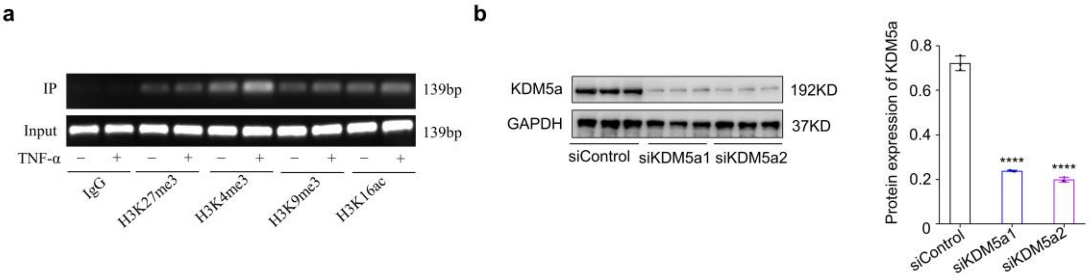

Supplementary Fig. 3 (a) ChIP-PCR results of WTAP promoter analysis, three independent experiments were repeated. **(b)** Knockdown efficiency of KDM5a in human NPCs using two specific siRNAs confirmed by western blot and RT-qPCR. Data are shown as the mean ± SD from three independent experiments, p<0.0001, p<0.0001, ****P<0.001, two-tailed unpaired Student's t-test.

Figure 4

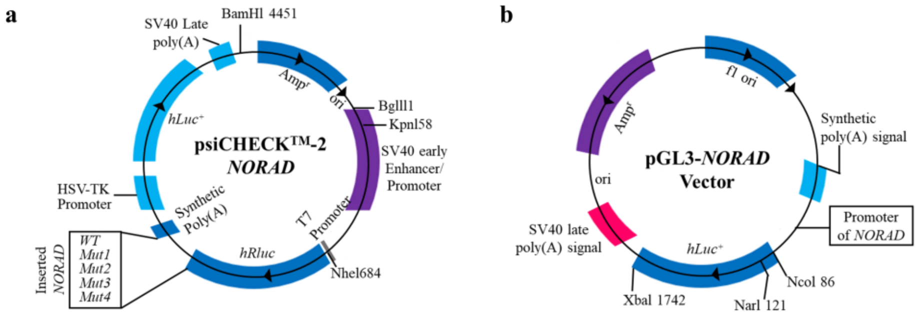

Supplementary Fig. 4 (a) Structure of psiCHECK™ luciferase reporter in which the wild-type or mutant NORAD sequence were cloned to analyze the transcription effect of m^{6}A on NORAD luciferase reporter assays. **(b)** Structure of pGL3 luciferase reporter in which the promoter region of NORAD was cloned to analyze the transcription effect of m^{6}A on NORAD luciferase reporter assays.

Figure 5

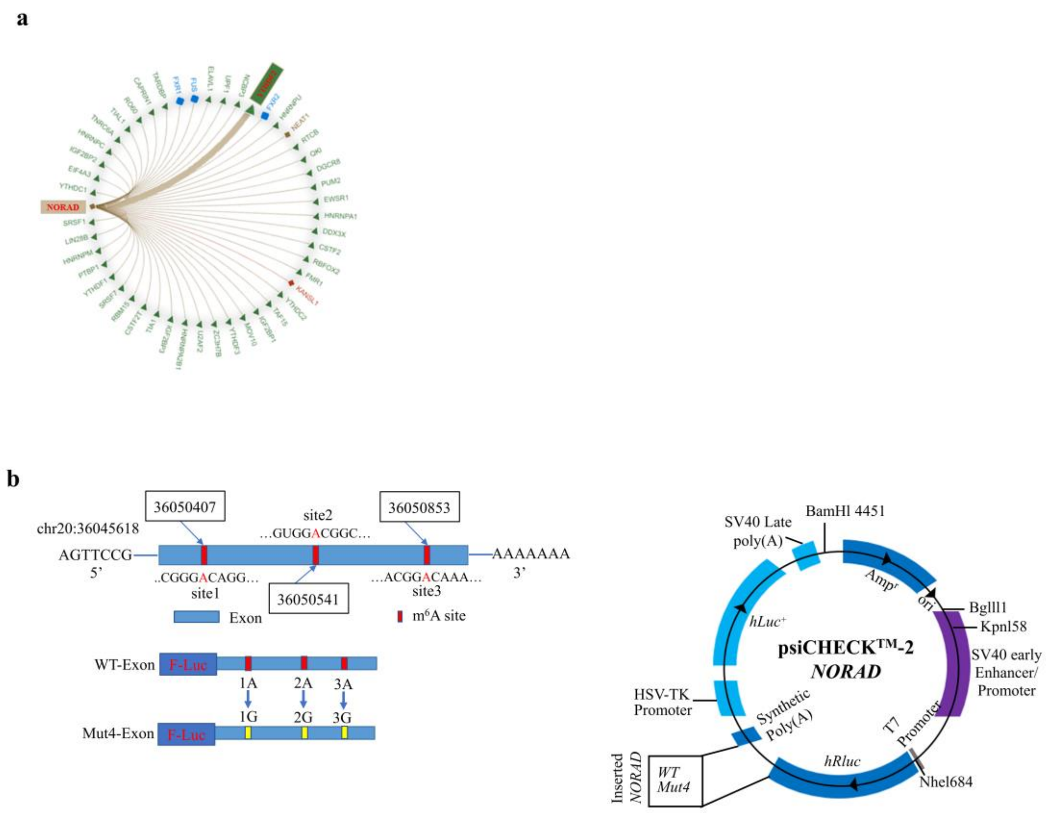

Supplementary Fig. 5 (a) Prediction of YTHDF2 interaction with NORAD by RNA interaction Database *RNA Intern*. **(b)** All m^{6}A sites mutation (Mut4) of NORAD and structure of psiCHECK^{TM} luciferase reporter in which the wild-type or Mut4 NORAD sequence were cloned.

Figure 6

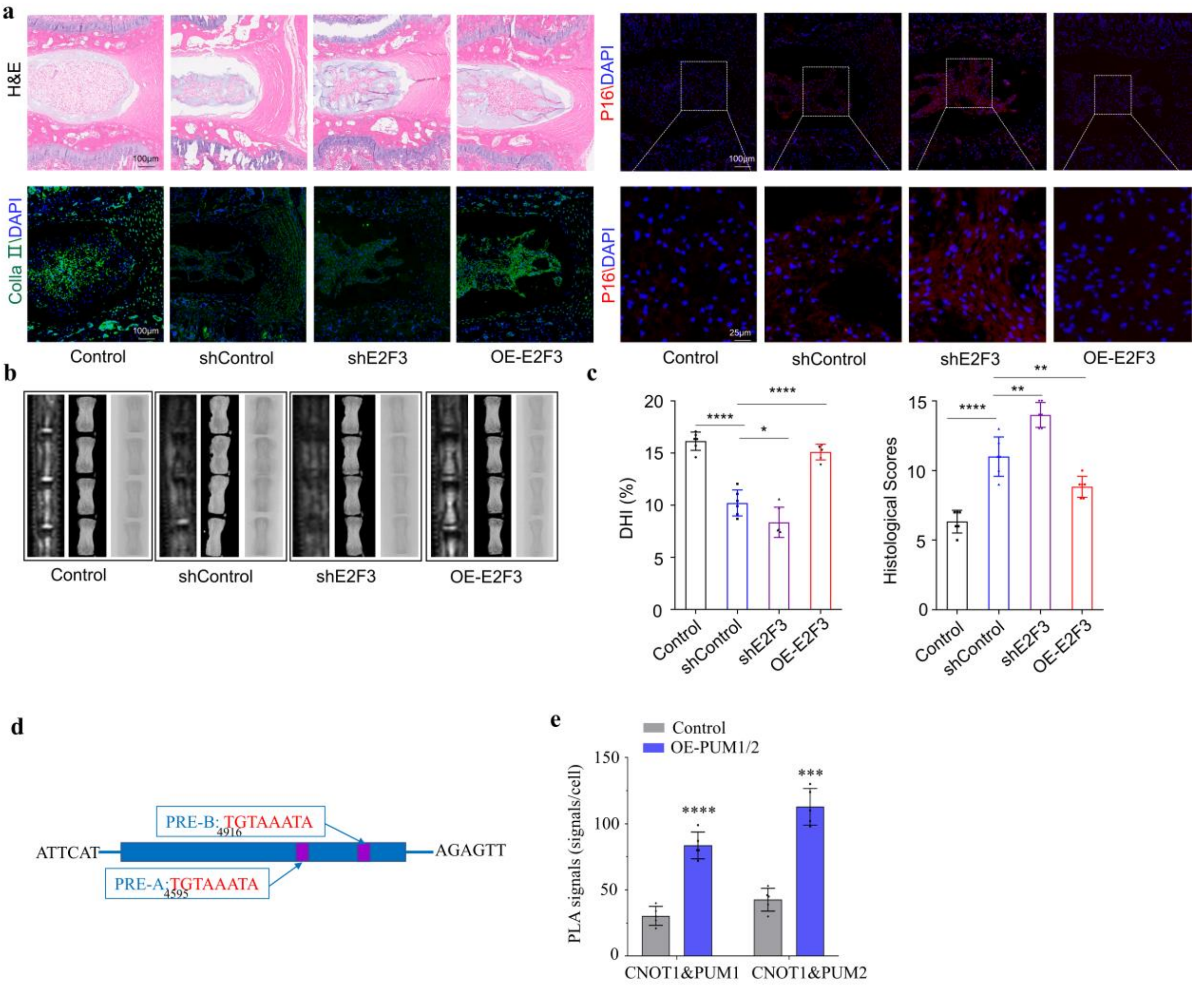

Supplementary Fig. 6 (a) Histological analysis of surgically-induced IVDD using AAV containing shcontrol, shE2F3 and OE-E2F3, analysed by HE and IF staining. **(b)** Radiographic presentation of IVDD model by MRI, μCT and X-Ray. **(c)** DHI% analysis (p<0.0001, p=0.0386, p<0.0001) and histological score (p<0.0001, p=0.0014, p=0.0078) of IVDD model; Data are presented as mean ± SD, n=6, **P*<0.05, *****P*<0.0001; n=6, ***P*<0.01, ****P*<0.001, two-tailed unpaired Student's t-test. **(d)** Two PRE sites in the 3'UTR of E2F3 transcript. **(e)** Number of PLA signals in PUM1 (p<0.0001) and PUM2 (p<0.0001) overexpresion cell. Data of signals were shown as the mean ± SD, n=5, ****p<0.0001, two-tailed unpaired Student's t-test.

Figure 7

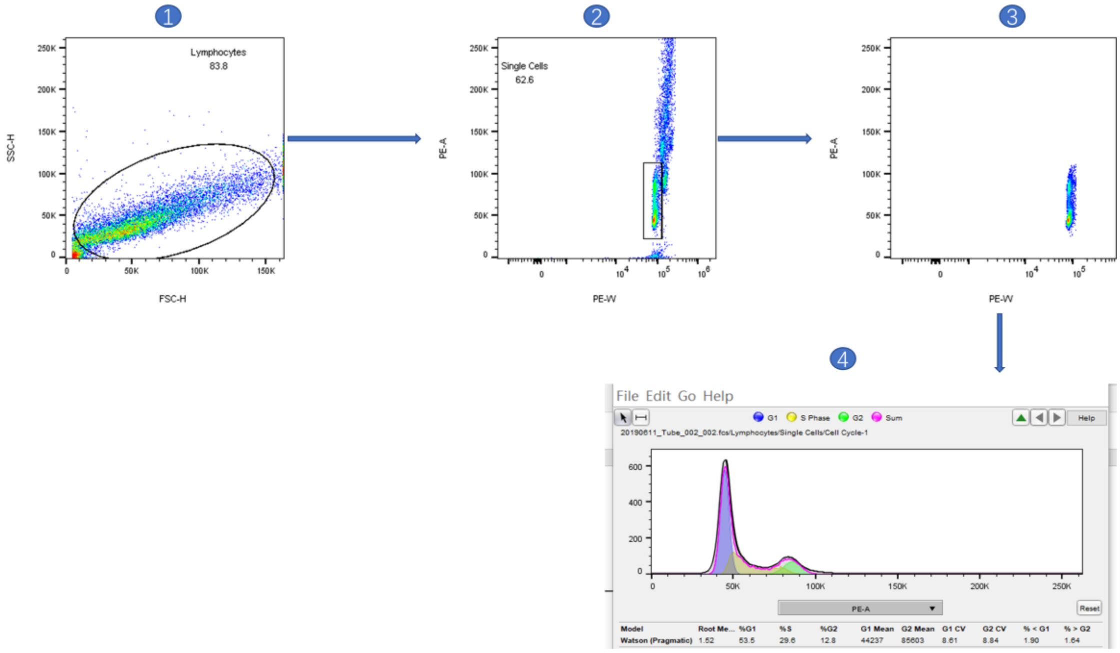

Supplementary Fig. 7 Representative plots showing gating strategy of NPCs during cell cycle analysis.

Supplementary Tables

Supplementary Table 1. **Antibodies used in this study**

Product name	Catalog No.	Manufacturer	Dilution Ratio
anti-P21	#2947	CST	1:1000
anti-P16	#80772	CST	1:1000
anti-P53	#2527	CST	1:1000
anti-METTL3	ab195352	Abcam	1:1000
anti-METTL14	ab220030	Abcam	1:1000
anti-WTAP	ab195380	Abcam	1:1000
anti-FTO	ab126605	Abcam	1:1000
anti-ALKBH5	ab195377	Abcam	1:1000
anti-GAPDH	60004-1-Ig	Proteintech	1:10000
anti-KDM5A	ab194286	Abcam	1:1000
anti-H3K4me3	ab213224	Abcam	1:1000
anti-DCP1a	ab183709	Abcam	1:1000
anti-IGF2BP1	22803-1-AP	Proteintech	1:500
anti- IGF2BP2	11601-1-AP	Proteintech	1:1000
anti- IGF2BP3	14642-1-AP	Proteintech	1:1000
anti-YTHDF1	17479-1-AP	Proteintech	1:1000
anti-YTHDF2	24744-1-AP	Proteintech	1:5000

anti-LAP2	ab185718	Abcam	1:500
anti-PUM1	NB100-259	Novus	1:1000
anti-PUM2	ab92390	Abcam	1:5000
anti-Ki67	#9449	CST	1:1000
anti-E2F3	27615-1-AP	Proteintech	1: 1000
anti-CNOT1	66507-1-Ig	Proteintech	1:500
anti-P16	ab211542	Abcam	1:100
Anti-Collagen II	ab34712	Abcam	1:100

***Supplementary Table 2.* Primers sequences**

Homo NORAD	Forward	5′- AGCGAAGTCCCGAACGACGA -3′
	Reverse	5′- TGGGCATTTCCAACGGGCCAA -3′
Homo MML1	Forward	5′- CACTTCACGGGGCGAACAT -3′
	Reverse	5′- GTGGCTTGCTGAAACGTAGC -3′
Homo MML2	Forward	5′-TCGCAAGCATAAGACGACCC -3′
	Reverse	5′-TTCCAGCTGCCAGTCTGATG -3′
Homo MML3	Forward	5′- TGCTTACAGCTGCAGGACTC -3′
	Reverse	5′- ACCCGGTAGGACAAATACTGG -3′
Homo MML4	Forward	5′- AATTAAACTGGAGGGCCCCG -3′
	Reverse	5′- GCAGGTATCACCTCGTCGG -3′
Homo MML5	Forward	5′- TTGGGGGTCGAGCACATAAC -3′
	Reverse	5′- TGCATCCACAAACCCATGAGA -3′
Homo KDM5a	Forward	5′- CTACAACAGGCTCGGTGGTT -3′
	Reverse	5′- ATGCGGTCCACCATTGTCAT -3′
Homo KDM5b	Forward	5′- TCAGGATAACCGGCTGCATT -3′
	Reverse	5′- AACGTATTGGCAAGGGCAGA -3′
Homo KDM5c	Forward	5′- GGTGGGAATGGGACACCAAA -3′
	Reverse	5′-GGGTTGCAAATGACCACACA -3′

Homo KDM5d	Forward	5′- GCCTTAGTAAGCAATGTAACACACA -3′
	Reverse	5′- AGGCTCTGGATCAGGCTGTA -3′
Homo E2F3	Forward	5′- CCCCGATTATTTTTGGCCCC -3′
	Reverse	5′- CTAGCTCCAGCCTTCGCTTT-3′
Mus-NORAD	Forward	5′-ACTGAGAGAGTGACTAACTGACCA-3′
	Reverse	5′-GATTCAAAGTACCCTGACAAGCAC-3′

***Supplementary Table 3*. Primers used for CLIP/ChIP/Pull down**

CLIP: Homo NORAD	Forward	5′- GCCATTGGGCGAGACCTACCT -3′
	Reverse	5′- GTTCGGGACTTCGCTCACCTT-3′
CLIP: Homo GAPDH	Forward	5′- GTGGACCTGACCTGCCGTCT -3′
	Reverse	5′- GGAGGAGTGGGTGTCGCTGT-3′
me-RIP: Homo NORAD	Forward	5′- GTCCTGACGACAACGGACAA -3′
	Reverse	5′- GGAGGAGTGGGTGTCGCTGT-3′
me-RIP: Homo HPRT1	Forward	5′- CCCTGGCGTCGTGATTAGTG-3′
	Reverse	5′- TCGAGCAAGACGTTCAGTCC-3′
Pull-down:T7-NORAD1	Forward	5′- TAATACGACTCACTATAGGGAGACCACCCTCTGGGAAGATTTACTG-3′
	Reverse	5′- ATTTAGGTGACACTATAGAAGGGAACAGGTGATTTGGCCATTCCCC -3′
CHIP:Homo WTAP	Forward	5′- ACGTTCTCCAGGTAACACCG -3′
	Reverse	5′- TTGCTAACTGGAACACGGGG -3′

***Supplementary Table 4*. siRNA and shRNA**

Sequence name	Target-position	oligo-sequence
homo-siControl	NA	5′- UUCUCCGAACGUCACGUUU -3′
homo-siWTAP1	348-370	5′-UUAAGAUCUGUGUACUUGCCC-3′
homo-siWTAP2	17642-17664	5′-UAUUUUGUAUAAUUACACCGU-3′
homo-siKDM5a	501-523	5′-AAAAUUUUGCUAGUUGAUCCA-3′
homo-siYTHDF2-1	249-271	5′- UUAAUCCAUCCUUUUGAUGUA-3′
homo-siYTHDF2-2	1875-1897	5′- UUUUAACACUUUCUUCUUCCU-3′
homo-siE2F3	197-219	5′- UUUCUUUUUAUUUUUCCGCAC-3′
homo-shNORAD	667-689	5′- UCAUUCUACCAUUUCUCUCUU-3′
homo-shPUM1	1050-1070	5′- GGUCCAAACCAGAAUGGUUCU-3′
homo-shPUM2	718-738	5′- UCGAUUUAGGAAGGGAAAUUU-3′
Mus-shE2F3	1368-1388	5′- GAGAGCCUACAAAUCCAUUUG-3′
Mus-shControl	NA	5′-ACUCAAAAGGAAGUGACAAGA-3′
Mus-shPUM1	4249-4271	5′- UUGUAUGAACAAAGUAAACUG-3′
Mus-shPUM2	3772-3794	5′- UUAGAAGUAUUUCAUAAUGUU-3′

Supplementary References

1 Li, Z. H. *et al.* N-6-methyladenosine regulates glycolysis of cancer cells through PDK4. *Nature Communications* **11**, doi:10.1038/s41467-020-16306-5 (2020).

2 Li, G. *et al.* Bone-derived mesenchymal stem cells alleviate compression-induced apoptosis of nucleus pulposus cells by N6 methyladenosine of autophagy. *Cell Death Dis* **11**, 103, doi:10.1038/s41419-020-2284-8 (2020).

3 Roberts, T. C. *et al.* Quantification of nascent transcription by bromouridine immunocapture nuclear run-on RT-qPCR. *Nat Protoc* **10**, 1198-1211, doi:10.1038/nprot.2015.076 (2015).

4 Ji, M. L. *et al.* Preclinical development of a microRNA-based therapy for intervertebral disc degeneration. *Nat Commun* **9**, 5051, doi:10.1038/s41467-018-07360-1 (2018).
