# Supplementary material for: WTAP-mediated m6A modification of lncRNA NORAD promotes intervertebral disc degeneration
Source: Nat Commun. 2022 Mar 18;13:1469. doi: 10.1038/s41467-022-28990-6 (PMC8933458; doi:10.1038/s41467-022-28990-6)
Supplement: Supplementary file 2 — Reporting Summary [file 41467_2022_28990_MOESM2_ESM.pdf]

nature portfolio

Corresponding author(s): Kun Wang; Cao Yang

Last updated by author(s): Nov 8, 2021

Reporting Summary

Nature Portfolio wishes to improve the reproducibility of the work that we publish. This form provides structure for consistency and transparency in reporting. For further information on Nature Portfolio policies, see our Editorial Policies and the Editorial Policy Checklist.

Statistics

For all statistical analyses, confirm that the following items are present in the figure legend, table legend, main text, or Methods section.

n/a	Confirmed	
☐	☒	The exact sample size (*n*) for each experimental group/condition, given as a discrete number and unit of measurement
☐	☒	A statement on whether measurements were taken from distinct samples or whether the same sample was measured repeatedly
☐	☒	The statistical test(s) used AND whether they are one- or two-sided<br>*Only common tests should be described solely by name; describe more complex techniques in the Methods section.*
☒	☐	A description of all covariates tested
☐	☒	A description of any assumptions or corrections, such as tests of normality and adjustment for multiple comparisons
☐	☒	A full description of the statistical parameters including central tendency (e.g. means) or other basic estimates (e.g. regression coefficient) AND variation (e.g. standard deviation) or associated estimates of uncertainty (e.g. confidence intervals)
☐	☒	For null hypothesis testing, the test statistic (e.g. *F*, *t*, *r*) with confidence intervals, effect sizes, degrees of freedom and *P* value noted<br>*Give P values as exact values whenever suitable.*
☒	☐	For Bayesian analysis, information on the choice of priors and Markov chain Monte Carlo settings
☒	☐	For hierarchical and complex designs, identification of the appropriate level for tests and full reporting of outcomes
☒	☐	Estimates of effect sizes (e.g. Cohen's *d*, Pearson's *r*), indicating how they were calculated

Our web collection on statistics for biologists contains articles on many of the points above.

Software and code

Policy information about availability of computer code

Data collection	Bio-rad CFX Connect Real-Time Systerm was used for PCR; ChemiDoc MP Imaging System (Bio-Rad, 12003154 Hercules, CA, USA).
Data analysis	Image J (V1.52v); Graphpad Prism 8.0.1 ; IGV; Image Lab(6.1); FlowJo V10; Stats R package (3.6.1); DESeq2 R-package

For manuscripts utilizing custom algorithms or software that are central to the research but not yet described in published literature, software must be made available to editors and reviewers. We strongly encourage code deposition in a community repository (e.g. GitHub). See the Nature Portfolio guidelines for submitting code & software for further information.

Data

Policy information about availability of data

All manuscripts must include a data availability statement. This statement should provide the following information, where applicable:

- Accession codes, unique identifiers, or web links for publicly available datasets
- A description of any restrictions on data availability
- For clinical datasets or third party data, please ensure that the statement adheres to our policy

The raw data from the Me-RIP-Seq analysis of NPCs have been deposited in the Gene Expression Omnibus database under the accession code GEO: “GSE169484 [https://www.ncbi. nlm.nih.gov /geo/query/acc.cgi?acc=GSE169484]”. The NGS data of single NPCs in this study are available under the accession identifier “GSE167931 [https://www.ncbi.nlm.nih.gov /geo/query/acc.cgi?acc=GSE167931]”. All other data supporting thefindings of this study are available within the article and its supplementary data. Source data are provided with this paper.

Field-specific reporting

Please select the one below that is the best fit for your research. If you are not sure, read the appropriate sections before making your selection.

☒ Life sciences ☐ Behavioural & social sciences ☐ Ecological, evolutionary & environmental sciences

For a reference copy of the document with all sections, see nature.com/documents/nr-reporting-summary-flat.pdf

Life sciences study design

All studies must disclose on these points even when the disclosure is negative.

Sample size	No statistical methods were used to predetermine sample size. The sample sizes were determined based on previous studies with similar experiments (Nature communications vol. 9,1 5051. 28 Nov. 2018; Nature communications vol. 12,1 5213. 3 Sep. 2021). For all the experiments, at least three separate experiments containing at least triplicate samples were performed, which allowed sufficient statistics to perform unpaired student ttest or ANOVA analysis, and gave p values to indicate the significance.
Data exclusions	No data was excluded.
Replication	All the experiments were replicated. In general, at least three separate experiments containing at least triplicate samples were performed determined based on the experiments.
Randomization	All samples was randomly assigned, and analyzed together in each experiment.
Blinding	The investigators were blinded to group allocation during data collection and analysis.

Reporting for specific materials, systems and methods

We require information from authors about some types of materials, experimental systems and methods used in many studies. Here, indicate whether each material, system or method listed is relevant to your study. If you are not sure if a list item applies to your research, read the appropriate section before selecting a response.

Materials & experimental systems

n/a	Involved in the study	
☐	☒	Antibodies
☒	☐	Eukaryotic cell lines
☒	☐	Palaeontology and archaeology
☐	☒	Animals and other organisms
☐	☒	Human research participants
☒	☐	Clinical data
☒	☐	Dual use research of concern

Methods

n/a	Involved in the study	
☒	☐	ChIP-seq
☐	☒	Flow cytometry
☒	☐	MRI-based neuroimaging

Antibodies

Antibodies used: The information of all the antibodies used in the study were listed in the Supplemental Table S1.

Validation: The validation information of all the antibodies could be searched in the manufacturer`s websites according to the source and identifier:
Human anti-P21 WB #2947 CST WB https://www.cellsignal.com/products/primary-antibodies/p21-waf1-cip1-12d1-rabbit-mab/2947
Human anti-P16 WB #80772 CST
https://www.cellsignal.com/products/primary-antibodies/p16-ink4a-d7c1m-rabbit-mab/80772
Mouse anti-P16 IF ab211542 Abcam
https://www.abcam.com/cdkn2ap16ink4a-antibody-epr20418-ab211542.html
Mouse Anti-Collagen II IF ab34712
https://www.abcam.com/collagen-ii-antibody-ab34712.html
Human anti-P53 WB /IF #2527 CST
https://www.cellsignal.com/products/primary-antibodies/p53-7f5-rabbit-mab/2527
Human anti-METTL3 WB/IP ab195352 Abcam
https://www.abcam.com/mettl3-antibody-epr18810-ab195352.html
Human anti-METTL14 WB/IP ab220030 Abcam
https://www.abcam.com/mettl14-antibody-cl4252-ab220030.html
Human anti-WTAP WB/IP ab195380 Abcam

https://www.abcam.com/wtap-antibody-epr18744-ab195380.html
Human anti-FTO WB ab126605 Abcam
https://www.abcam.com/fto-antibody-epr6894-ab126605.html
Human anti-ALKBH5 WB ab195377 Abcam
https://www.abcam.com/alkbh5-antibody-epr18958-ab195377.html
Human anti-GAPDH WB 60004-1-Ig Proteintech
https://www.ptglab.com/products/GAPDH-Antibody-60004-1-Ig.htm
Human anti-KDM5A WB ab194286 Abcam
https://www.abcam.com/kdm5a--jarid1a--rbbp2-antibody-epr18651-ab194286.html
Human anti-H3K4me3 WB/IP ab213224 Abcam
https://www.abcam.com/histone-h3-tri-methyl-k4-antibody-epr20551-225-chip-grade-ab213224.html
Human anti-DCP1a IF ab183709 Abcam
https://www.abcam.com/dcp1a-antibody-epr13822-ab183709.html
Human anti-IGF2BP1 WB 22803-1-AP Proteintech
https://www.ptglab.com/products/IGF2BP1-Antibody-22803-1-AP.htm
Human anti- IGF2BP2 WB 11601-1-AP Proteintech
https://www.ptglab.com/products/IGF2BP2-Antibody-11601-1-AP.htm
Human anti- IGF2BP3 WB 14642-1-AP Proteintech
https://www.ptglab.com/products/IGF2BP3-Antibody-14642-1-AP.htm
Human anti-YTHDF1 WB 17479-1-AP Proteintech
https://www.ptglab.com/products/YTHDF1-Antibody-17479-1-AP.htm
Human anti-YTHDF2 WB/IP 24744-1-AP Proteintech
https://www.ptglab.com/products/YTHDF2-Antibody-24744-1-AP.htm
Human anti-LAP2 WB ab185718 Abcam
https://www.abcam.com/lap2-antibody-ab185718.html
Human anti-PUM1 WB/IF NB100-259 Novus
https://www.novusbio.com/products/pum1-antibody_nb100-259
Human anti-PUM2 WB/IF ab92390 Abcam
https://www.abcam.com/pumilio-2-antibody-epr3813-ab92390.html
Human anti-Ki67 IF #9449 CST
https://www.cellsignal.com/products/primary-antibodies/ki-67-8d5-mouse-mab/9449
Human anti-E2F3 WB/IF 27615-1-AP Proteintech
https://www.ptgcn.com/products/E2F3-Antibody-27615-1-AP.htm
Human anti-CNOT1 IF/WB 66507-1-Ig Proteintech
https://www.ptglab.com/products/CNOT1-Antibody-66507-1-Ig.htm

Animals and other organisms

Policy information about studies involving animals; ARRIVE guidelines recommended for reporting animal research

Laboratory animals	Norad KO mice and C57 mice were raised in Animal Center of Tongji Medical College, Huazhong University of Science and Technology under SPF condition, with 12 hours of dark/light cycle, with 23°C ambient temperature and 50% humidity. At 8 weeks, male mice were choosen for experiments.
Wild animals	No wild animals were used in this study.
Field-collected samples	No samples were collected from field.
Ethics oversight	This study was approved by The Institutional Animal Care and Use Committee (IACUC) at Tongji Medical College, Huazhong University of Science and Technology (NO. S2394).

Note that full information on the approval of the study protocol must also be provided in the manuscript.

Human research participants

Policy information about studies involving human research participants

Population characteristics	NP tissues were obtained from 68 patients (38 females and 30 males; age 54.2 ± 8.4 years) with degenerative disc disease undergoing surgery. The control samples were taken from 71 patients (42 females and 29 males; age 25.2 ± 14.2 years) undergoing surgery due to scoliosis or thoracolumbar fracture after informed consent was obtained.
Recruitment	The patients' medical records were also collected and magnetic resonance images were used to evaluate the IVD degenerative level according to Pfirrmann MRI-grade system. The 68 patients (38 females and 30 males; age 54.2 ± 8.4 years) with degenerative disc disease undergoing surgery and 71 patients (42 females and 29 males; age 25.2 ± 14.2 years) undergoing surgery due to scoliosis or thoracolumbar fracture were recruited.
Ethics oversight	This study was approved by the Ethics Committee of Tongji Medical College, Huazhong University of Science and Technology (NO. S341)

Note that full information on the approval of the study protocol must also be provided in the manuscript.

Flow Cytometry

Plots

Confirm that:

☒ The axis labels state the marker and fluorochrome used (e.g. CD4-FITC).

☒ The axis scales are clearly visible. Include numbers along axes only for bottom left plot of group (a 'group' is an analysis of identical markers).

☒ All plots are contour plots with outliers or pseudocolor plots.

☒ A numerical value for number of cells or percentage (with statistics) is provided.

Methodology

Sample preparation	After silencing of NORAD or PUM1/2 and the silencing efficiency was tested, nucleus pulposus cells were treated with serum starvation process. And then cells were collected, fixed and stained using Cell CYcle and Apoptosis Analysis Kit (Beyotime, C1052, Shanghai, China)
Instrument	Samples were sorted using the FACS (BD FACS Calibur; BD Biosciences, San Jose, CA, USA) .
Software	Data were analyzed using flowjo V10 (BD biosciences, Franklin Lakes, New Jersey).
Cell population abundance	Nucleus pulposus cells without any staining were used as control.
Gating strategy	The cell cycle analysis were performed using flowjo V10 (BD biosciences, Franklin Lakes, New Jersey) with X-axis was chosen FL2-W while Y-axis was chosen FL2-A.

☒ Tick this box to confirm that a figure exemplifying the gating strategy is provided in the Supplementary Information.
